# Tapered Optical Fiber Optofluidics: Bridging In-Fiber and Outside-Fiber Architectures Toward Autonomous Lab-on-Fiber Biosensing

**DOI:** 10.3390/s25175229

**Published:** 2025-08-22

**Authors:** Alba Lako, Marzhan Sypabekova

**Affiliations:** Department of Bioengineering, Civil Engineering, and Environmental Engineering, U.A. Whitaker College of Engineering, Florida Gulf Coast University, Fort Myers, FL 33965, USA

**Keywords:** biosensors, microfluidics, optofluidics, lab-on-a-fiber, tapered optical fibers

## Abstract

Optical fiber-based biosensors have proven to be a powerful platform for chemical and biological analysis due to their compact size, fast response, high sensitivity, and immunity to electromagnetic interference. Among the various fiber designs, tapered optical fibers have gained prominence due to the increased evanescent fields that significantly improve light–analyte interactions, making them well-suited for advanced sensing applications. At the same time, advances in microfluidics have allowed for the precise control of small-volume fluids, supporting integration with optical fiber sensors to create compact and multifunctional optofluidic systems. This review explores recent developments in optical fiber optofluidic sensing, with a focus on two primary architectures: in-fiber and outside-fiber platforms. The advantages, limitations, and fabrication strategies for each are discussed, along with their compatibility with various sensing mechanisms. Special emphasis is placed on tapered optical fibers, focusing on design strategies, fabrication, and integration with microfluidics. While in-fiber systems offer compactness and extended interaction lengths, outside-fiber platforms offer greater mechanical stability, modularity, and ease of functionalization. The review highlights the growing interest in tapered fiber-based optofluidic biosensors and their potential to serve as the foundation for autonomous lab-on-a-fiber technologies. Future pathways for achieving self-contained, multiplexed, and reconfigurable sensing platforms are also discussed.

## 1. Introduction

Optical fiber-based biosensors have become one of the newest tools for chemical and biomedical analysis, offering advantages in remote sensing, fast response, and immunity to electromagnetic interference [1]. In such sensors, light guided in a fiber probe interacts with surrounding analytes, allowing detection via changes in intensity, phase, or wavelength. Many geometrically modified optical fibers, including tapered optical fibers [2,3,4,5,6,7], side-polished optical fibers [8,9,10,11], D-shaped optical fibers [12,13,14,15,16,17], and U-bent optical fibers [18], have been used to increase the evanescent field of the guided core or cladding modes. The interaction of the evanescent field with the surrounding environment increases the sensitivity of the biosensor. Optical fibers have been employed with a variety of sensing mechanisms, including Surface Plasmon Resonance (SPR), Raman scattering, and Whispering-Gallery Modes (WGMs), among others. These diverse transduction principles allow for flexible and highly sensitive detection strategies. A key factor in the performance of fiber-based sensors is surface functionalization, which not only allows for specific analyte recognition but also significantly improves sensitivity. Common approaches include the deposition of metallic layers, such as gold or silver, and the use of nanomaterials like graphene oxide (GO) to amplify the optical response. Additionally, biomolecular probes such as aptamers and antibodies are frequently immobilized on the fiber surface to facilitate selective detection. The versatility in structure, compatibility with multiple sensing mechanisms, and tunable surface chemistry make optical fibers highly suitable for a wide range of biological and chemical sensing applications.

Microfluidic chips are another emerging technology that are used to precisely manipulate small fluid volumes (from nanoliters to microliters) through networks of channels, allowing for the delivery to the sensor in a controlled manner [19]. Integrating fibers with microfluidics (often called optofluidics) yields compact sensing systems that incorporate both sample handling and optical detection simultaneously [20,21]. Additionally, the channel sizes of microfluidic chips are usually tens to hundreds of microns, which provide light and fluid confinement on the micro-scale and ensure enhanced light–analyte interactions [22]. Furthermore, microfluidic devices offer control of small-volume fluids, thereby greatly reducing sample consumption. This integration is especially valuable for biosensing, where rapid, on-site testing is desired. Ultimately, the goal of optofluidic devices is to advance toward fully self-contained lab-on-a-fiber platforms capable of autonomous, high-sensitivity analysis outside of laboratory settings.

Within this context, tapered optical fibers have gained prominence for boosting biosensor performance. A tapered fiber is a standard optical fiber that has been locally narrowed (usually to a few-micron or sub-micron waist diameter) over some length, exposing a portion of the guided light as an evanescent field outside the fiber [23,24]. This strong evanescent field interacts directly with the surrounding medium, dramatically increasing sensitivity to the refractive index (RI) changes or absorption by analytes near the fiber surface [1]. Tapered fiber sensors are also extremely small and flexible, which facilitates integration into miniaturized devices and even in vivo or in situ measurements in otherwise inaccessible locations [25,26]. Researchers have identified tapered fiber biosensors as a promising alternative to conventional SPR sensing [27,28,29,30] or fiber Bragg grating sensors [31], offering simplicity and comparable (or better) sensitivity [25]. The combination of tapered fiber optics with microfluidic chips—optofluidic tapered fiber biosensors—combines the strengths of both technologies, yielding sensors that are highly sensitive, compact, and capable of real-time analysis with minimal sample volume [19].

**Figure 1:**
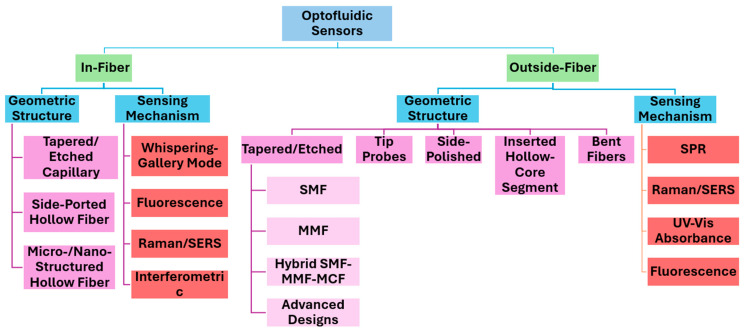
Schematic overview of the structure and content of this review.

The following sections are a comprehensive review of recent advancements in optical fiber-based optofluidic systems, with a particular focus on tapered fiber optofluidic platforms (Figure 1). The discussion first addresses the two primary integration strategies: in-fiber and outside-fiber platforms. For each category, the fabrication materials and methods are examined, highlighting their respective advantages and limitations. These platforms are further classified into subcategories based on distinctive fiber structures and sensing mechanisms, providing an analysis of their operational methods and applications, benefits and challenges, and potential for integration into autonomous lab-on-a-fiber systems. Then a structured review of recent studies on tapered fiber geometries is provided, as well as materials and fabrication techniques, along with a discussion of their integration within outside-fiber microfluidic environments. The benefits, challenges, and future directions of combining tapered fibers with optofluidic systems are explored, highlighting their potential in the development of next-generation lab-on-a-fiber technologies. To contextualize the advantages and limitations of fiber-based biosensors, we also included a concise comparison with non-fiber Microfluidic Platforms.

## 2. Optofluidic Integration Strategies: In-Fiber vs. Outside-Fiber Architectures

Microfluidic devices are small-scale systems designed to precisely control fluids within channels that range from tens to hundreds of micrometers. These systems have been a key part of lab-on-a-chip platforms, which combine sample preparation and analysis into one integrated, automated device [32,33]. In biosensing applications, lab-on-a-chip platforms are essential for tasks like mixing reagents, filtering samples, culturing cells, and delivering analytes directly to sensor surfaces, while minimizing reagent usage and speeding up processing times [34,35]. The integration of optical fibers with microfluidics, known as optofluidics, uses the precise fluid handling capabilities of microfluidic systems to improve the performance of optical fiber-based sensors. When all preparation/detection steps are hosted on the same substrate as the fiber sensor, the system is often termed a lab-on-a-fiber. We classified optofluidic devices into two categories based on two practical configurations: in-fiber optofluidics, where the fluid flows inside the fiber, and outside-fiber optofluidics, where the fiber probe sits in an external microfluidic chip that handles fluid flow. To guide the discussion that follows, we further organized the literature within each configuration. In-fiber platforms are grouped into three sub-families: (i) tapered or chemically etched capillary sensors, (ii) side-ported hollow-fiber sensors, and (iii) micro-/nano-structured hollow fibers. Outside-fiber systems are divided by their dominant optical transduction schemes: (i) SPR fiber-chip platforms, (ii) Raman/Surface Enhanced Raman Scattering (SERS) fiber-chip platforms, (iii) ultraviolet-visible (UV–Vis) absorbance devices, and (iv) fluorescence-based fiber-chip sensors.

## 3. In-Fiber Optofluidics

### 3.1. Flow Principles and Materials

**Figure 2:**
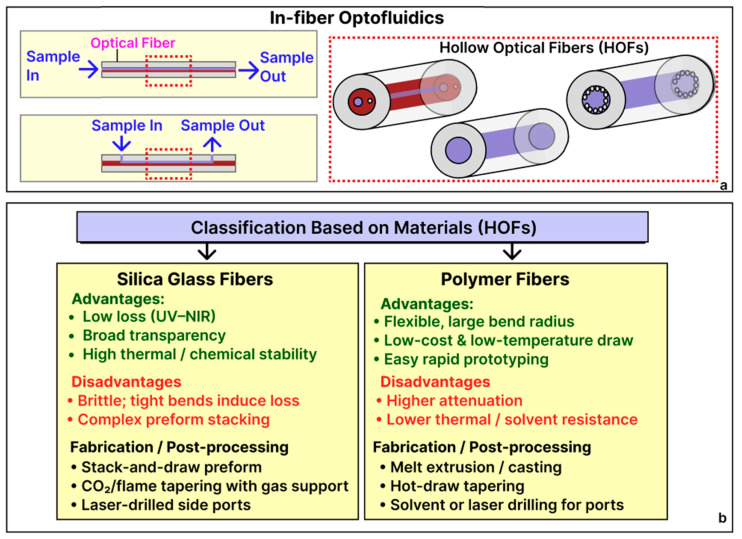
In-fiber optofluidics principle and material-driven design. (**a**) Principle of in-fiber optofluidics: the guided light and the sample fluid (blue) travel through a common hollow or microstructured region of the fiber; inlet/outlet ports enable side openings for controlled flow. (**b**) Material-based comparison of silica glass and polymer optical fibers: key advantages, drawbacks, and typical fabrication or post-processing techniques.

In in-fiber optofluidic devices, the guided optical light and fluid analytes share the same hollow or microstructured region within the fiber (Figure 2a). The extended interaction length inside these fluidic fiber cores improves the sensitivity by increasing the analyte–light contact area, while simultaneously allowing the fiber to function directly as a microfluidic channel, thereby simplifying device integration and analyte detection [36]. Until now, researchers have optimized these designs by tailoring the diameter of the fiber and internal fiber geometries, such as hole patterns and cladding structures, to manage fluid flow resistance, enhance the evanescent field overlap with the analyte, introduce resonant optical cavities, and reduce transmission losses [37].

Material choice for these fibers is predominantly dictated by optical transparency and mechanical needs (Figure 2b). Silica glass fiber is extensively used due to its low optical attenuation and broad transparency range. Alternatively, polymer optical fibers (POFs) offer mechanical flexibility, easier processing, and reduced costs, although typically at the expense of higher optical losses [36]. Fabrication of in-fiber optofluidic sensors typically begins at the preform stage. Microstructured or hollow-core geometries are commonly fabricated through precision methods such as stack-and-draw or extrusion techniques, resulting in silica or polymer hollow optical fibers (HOFs) featuring accurately controlled air–hole lattices (Figure 2a, inset). Subsequent fiber tapering, performed by laser or flame heating under controlled internal gas pressure, refines the core size and dramatically improves the optical field–analyte interaction. The fluid in such in-fiber optofluidic systems can be introduced from one end of the fiber and exit from the other, directed by external pump systems. In addition, fluid access ports and side openings can be created by CO_2_ laser micromachining or focused-ion-beam milling to initiate fluid introduction.

### 3.2. Tapered/Etched Capillary Sensors

In-fiber optofluidic devices have evolved significantly, demonstrating various performances through different design adaptations. Most work exploited a simple glass microcapillary HOF. To make such fiber sensitive for biosensing applications, the geometry was adjusted/modified. For example, flame (or CO_2_ laser) draw under a controlled internal gas pressure was used to taper a standard glass capillary down to a few microns, thereby increasing the evanescent field overlap with the flowing analyte and, when the wall is thin enough, supporting WGM resonances on the inner surface.

**Figure 3:**
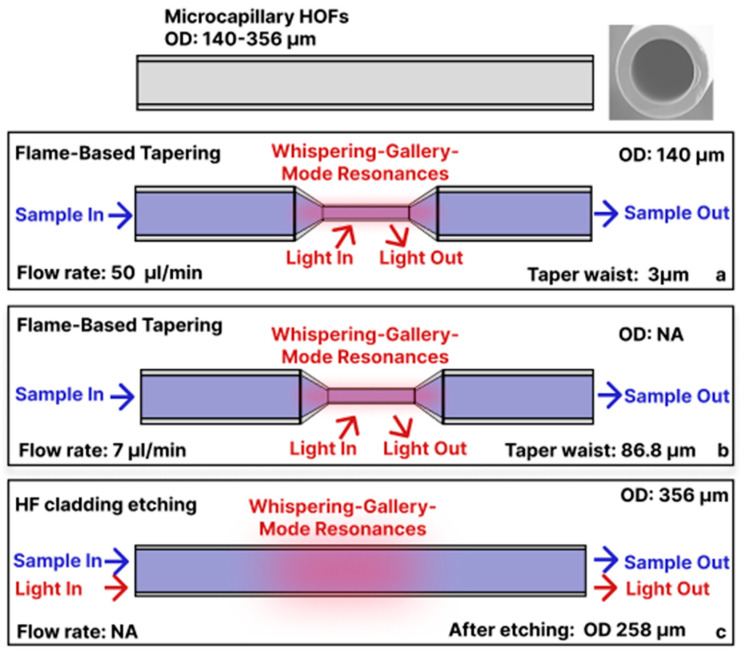
Schematic of microcapillary HOFs and three approaches to creating sensing regions: Flame-based tapering (**a**) to sub-micron waist (3 µm) and (**b**) to intermediate waist (86.8 µm) to induce WGM; (**c**) hydrofluoric acid (HF) cladding etching of the full fiber (258 µm) without tapering to induce WGM. The cross-section micrograph of the microcapillary HOF was adapted from Ref. [38]. © Elsevier.

Li et al. tapered a 140 µm-outer diameter (OD) silica tube to a 3 µm waist and used a syringe pump to flow 100 nm fluorescent polystyrene beads to detect fluorescence through the hollow core at 50 µL/min (Figure 3a) [38]. The excitation light at 400 nm was coupled into the tapered hollow core region to induce evanescent coupling by an external fiber, and fluorescence was measured by another fiber connected to a spectrometer. The amount of liquid in such a tapered HOF was 0.153 µL. Similar work was conducted by Wan et al., where they tapered the microcapillary HOF down to an 86.8 μm waist diameter to induce the WGM [39]. This in-fiber optofluidic device was used to detect different glucose concentrations via functionalization of the internal interface of the tapered HOF with a glucose oxidase enzyme. The detection setup was the same as in Li et al., i.e., requiring an external pump to flow liquid at 7 µL/min (sample volume: 90 nL inside the tapered HOFs and external fibers to excite and collect the signal (Figure 3b). The device was able to detect WGM shifts caused by glucose concentration variations down to 2.78 mM. Instead of tapering, Zhang et al. chemically etched a silica microcapillary HOF (OD 356 μm) to create a thin-walled HOF (OD 258 μm) and induce WGM for hemoglobin sensing [40]. Sample solutions were pushed through the fiber with a syringe or peristaltic pump (Figure 3c). A 532 nm laser excited rhodamine 6G in the fiber wall; hemoglobin quenched this fluorescence, so the laser emission dropped in proportion to Hb concentration, achieving a limit of detection (LOD) of 0.7 nM. Laser pumping and signal collection via spectrometer were both performed laterally on the same fiber segment.

### 3.3. Side-Ported Hollow-Fiber Sensors

**Figure 4:**
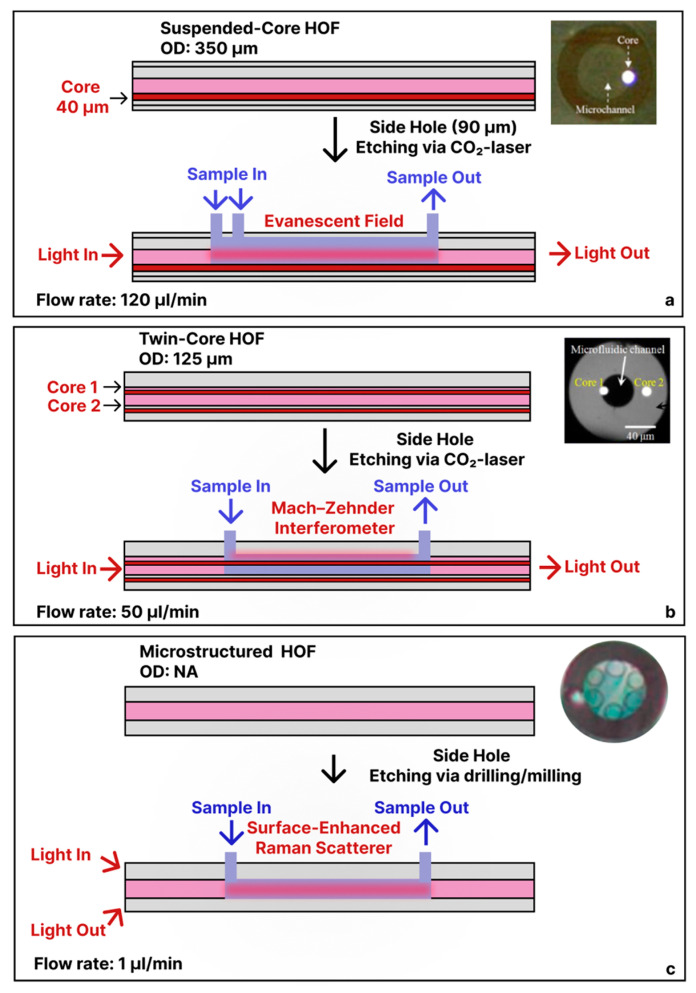
In-fiber optofluidic sensing concepts based on HOFs micro-machined with side holes that serve as fluidic inlet and outlet ports. (**a**) Suspended-core HOF; (**b**) twin-core HOF; and (**c**) microstructured HOF. Cross-section micrographs of each fiber type adapted in (**a**–**c**), reproduced with permission from Refs. [41,42,43]. © Elsevier; ©MDPI, licensed under CC BY 4.0, respectively.

Extending beyond tapered/etched microcapillaries, where the fluid enters and exits the fiber laterally, in-fiber optofluidic devices were fabricated by adding specially structured inlet and outlet ports along the fiber. For example, Yang et al. used a 350 µm-OD suspended-core HOF (containing a 40 µm solid core), then etched two ≈ 90 µm-wide side holes (via CO_2_ laser) for two reagent inlets 1 cm apart and one outlet 5 cm downstream [41]. By co-flowing rhodamine 6G and nitrite streams at a combined 120 µL/min via an injection pump, the evanescent field of the suspended core both excited fluorescence and recoupled the emission into the waveguide (Figure 4a). In this setup, the exciting light was focused and coupled into one end of the optical fiber, providing enough length of the core for the excitation and the collection of the fluorescence at another end by a photomultiplier through a monochrometer. Another study demonstrated a 125 µm-OD twin-core HOF (42 µm bore with two 7.8 µm cores) fusion spliced to a multimode fiber (MMF)/single-mode fiber (SMF) and with side-drilled holes for fluidic inlets (Figure 4b), where core 1 served as the sensing arm and core 2 as the reference arm. With 50 µL/min sample flow via a syringe pump, streptavidin–biotin binding around the suspended core produced a Mach–Zehnder Interferometer shift with a minimum detectable range for streptavidin at ∼3 × 10^−3^ mg/mL [42]. Wang et al. demonstrated a microfluidic Raman detection system using a microstructured HOF [43]. The authors coupled a 785 nm laser into the HOF via solid core and collected the backward-scattered Raman signal using a spectrometer. The in-fiber optofluidic device could accommodate a 3.7 μL solution and it was delivered via drilled side holes into the central channel using the syringe pump at 1 µL/min (Figure 4c). Their design allowed the detection of ethanol down to 0.32 g/L and glucose at concentrations as low as 10 g/L. Adamu et al. developed an in-fiber optofluidic device similarly by using focused-ion-beam milling on the outer cladding to create a precise, 50 µm-deep fluidic channel [44]. The milled channel, positioned between adjacent 500 nm thin capillary tubes separated by a 2.8 µm gap, enabled direct fluid access to the hollow core. Their work focused on the fabrication approach rather than specific sensing applications.

### 3.4. Micro-/Nano-Structured Hollow Fibers

**Figure 5:**
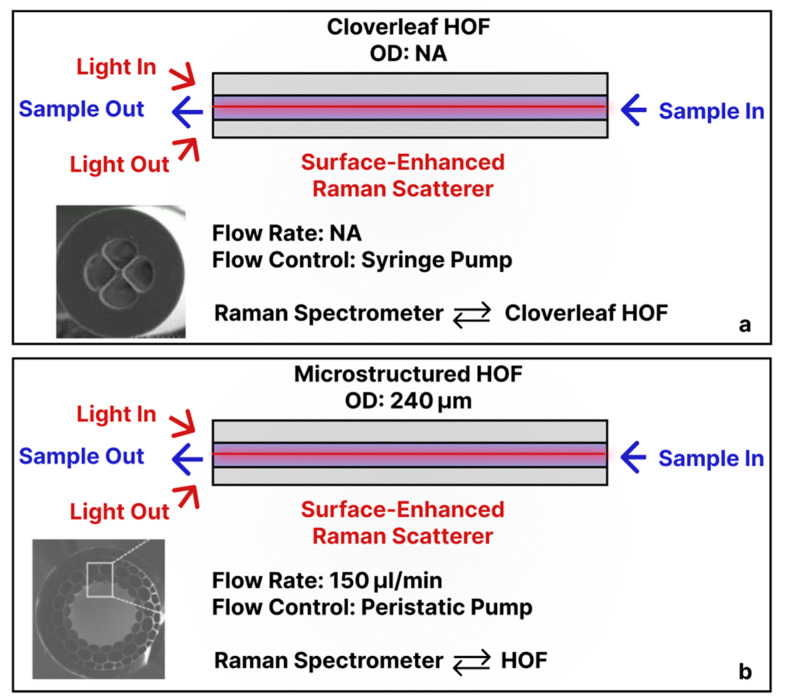

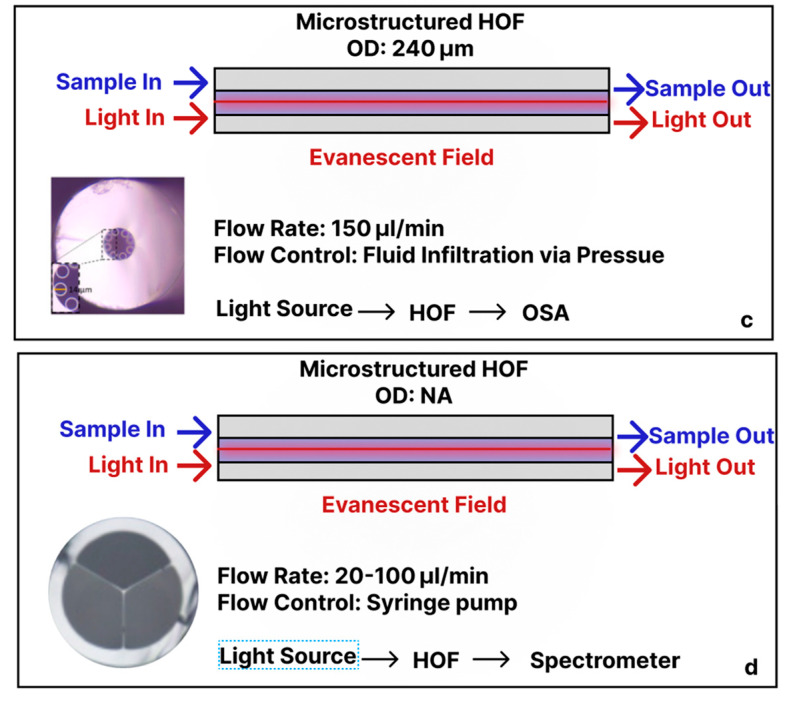
Representative in-fiber optofluidic biosensor geometries. (**a**) Cloverleaf HOF; (**b**) microstructured HOF. (**c**) Pressure-infiltrated microstructured HOF; (**d**) tri-channel microstructured HOF. Cross-section micrographs of each fiber type adapted from and reproduced, respectively, with permission from Refs. [45,46,47,48] © IEEE, MDPI (CC BY 4.0), and ©Optica Publishing Group (CC BY 4.0).

Other in-fiber optofluidics-based biosensors employed alternative internal fiber geometries. For example, Wang et al. demonstrated a cloverleaf HOF that contained four longitudinal channels acting as microfluidic conduits (Figure 5a). The inner walls of those channels were functionalized with silver nanoparticles (AgNPs: d ≈ 80 nm) to promote SERS [45]. Raman signals from the sample were excited by a 785 nm laser propagating through the fiber core, and the SERS response was coupled back for detection. With the flow driven by a syringe pump, the device detected Rhodamine 6G with an LOD of 1 pM. Merdalimova et al. built a similar SERS platform on a 240 µm-OD microstructured HOF (Figure 5b). Liquid was pulled through the large central hollow core with a peristaltic pump; that same channel served as both the microfluidic pathway and the optical waveguide. The authors coated the core surface with gold nanoparticles (AuNPs: d ≈ 8 nm), and the resulting nanostructured layer generated SERS when excited by a broadband light source, with signals collected by a spectrometer [46]. Extending the concept to DNA diagnostics, Khozeymeh et al. used a tube-lattice HOF whose interior was functionalized with peptide nucleic acid probes (Figure 5c) [47]. As DNA-containing solutions flowed through the hollow core, hybridization events produced measurable shifts in the fiber’s transmission bands (LOD 5 μM). The fluid flow was controlled by an infiltration process via pressure. Moeglen-Paget et al. developed an HOF in which a solid silica core is supported by three large liquid-filled channels (Figure 5d). A 638 nm pump launched into the core excited fluorophores via the evanescent field extending into those channels. By coating the inner channel walls with Cy5-labeled anti-haptoglobin antibodies, the sensor achieved ≈ 10 pM sensitivity to haptoglobin. Reagents were introduced through a syringe pump connected to a hypodermic needle sealed onto one fiber end, so liquid traversed the three hollow channels [48].

## 4. Outside-Fiber Optofluidics

### 4.1. Flow Principles, Materials, and Optical Design Considerations

**Figure 6:**
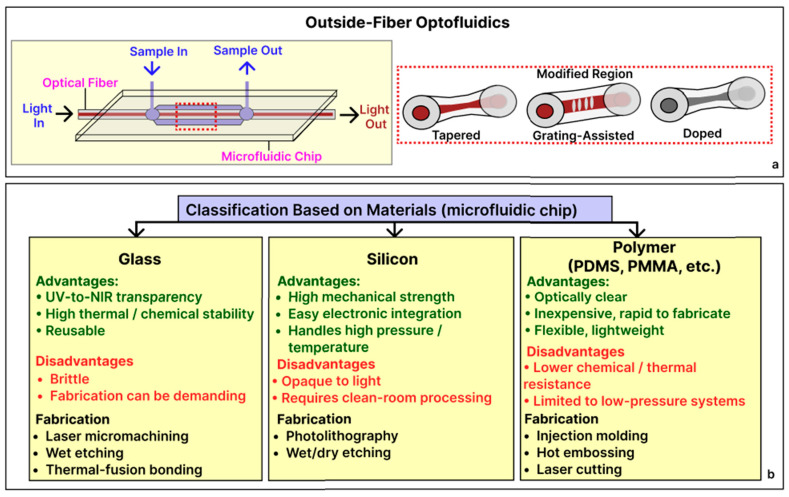
Outside-fiber optofluidic platforms and material selection. (**a**) Schematic of an outside-fiber device: an optical fiber passes through a microfluidic chip so that the guided mode interacts with the sample flowing (blue) in an adjacent channel; tapered, grating-assisted, or doped fiber sections can be inserted to enhance light–analyte coupling. (**b**) Material-based comparison of microfluidic substrates: glass, silicon, and polymers; principal advantages, disadvantages, and representative fabrication routes relevant to fiber-integrated biosensors.

By contrast, outside-fiber optofluidic architectures embed or align an optical fiber within a distinct microfluidic chip so that the liquid stream flows adjacent to the fiber rather than inside it (Figure 6a). This geometry supports “front-end separation, back-end detection”: analytes can be mixed, reacted, or purified on-chip immediately before optical interrogation, a sequence that enhances specificity and mitigates the matrix effect [19]. Automated, laminar delivery also ensures uniform exposure of the sensing region, thereby improving run-to-run reproducibility relative to manual sample injection [19,49].

In these systems, the primary materials are those used for the microfluidic substrate, not the fiber itself. Silicon microfluidic chips tolerate elevated pressures and temperatures and permit sub-micrometer lithographic features, yet their optical opacity, high cost, and strict clean-room fabrication requirements can limit widespread use [50]. Glass substrates are transparent from the ultraviolet through the near-infrared region and exhibit extremely low gas permeability, qualities ideally suited for optical biosensing and fluorescence assays, although their brittleness is a drawback in mechanically demanding environments [51]. Polymeric platforms, specifically elastomers such as polydimethylsiloxane (PDMS), and thermoplastics such as polymethyl methacrylate (PMMA) and poly vinyl carbonate (PVC), are inexpensive, flexible, and highly amenable to rapid prototyping [52]. PDMS and PMMA are especially popular because they easily form leak-tight seals around embedded fibers [25] and are biocompatible, though they swell in many organic solvents and are unsuitable for sustained high-pressure operations [53]; their widespread use is reflected in numerous optofluidic biosensing studies [54,55,56,57,58,59].

Fabrication routes of microfluidic chips can be dictated by the substrate material illustrated in Figure 6b. Glass devices are typically patterned by femtosecond or CO_2_ laser micromachining or by photolithography, followed by wet chemical etching; the structured wafer is then hermetically sealed to a cover plate by thermal-fusion bonding, yielding optically transparent and chemically durable chips that tolerate repeated use. Silicon chips depend on clean-room photolithography in combination with wet or deep reactive-ion etching, often preceded by thin-film deposition, and are finished by anodic or fusion bonding, producing sub-micrometer features capable of withstanding high pressures and temperatures [60,61]. Polymer platforms provide the most rapid and economical fabrication: elastomeric PDMS layers are cast by soft lithography, whereas rigid thermoplastics such as PMMA are mass produced by injection molding or hot embossing or quickly prototyped through laser cutting and high-resolution 3D printing [61]. Across all three material classes, optical fibers can be integrated by placing the fiber in a dedicated groove, and the chip is subsequently closed with thermal, solvent, or adhesive bonding, ensuring precise optical alignment and leak-tight fluid containment. Before that, optical fibers can be altered (tapered, grating inscribed, and coated or doped with nanoparticles) to enhance the sensitivity (Figure 6a, inset).

Alongside fluidic behavior and material choice, the operational wavelength regime and dispersion properties of the fiber strongly influence sensing performance. Material dispersion (set by fiber composition) and waveguide dispersion (set by core–cladding geometry) affect the light–analyte interaction and spectral resolution from UV to infrared (IR). Short wavelengths can suffer from Rayleigh scattering and autofluorescence, while long wavelengths offer greater evanescent field penetration. In narrowband schemes, dispersion is often addressed in calibration; in broadband approaches, it can impact fringe spacing and resolution. Choosing the spectral range to match analyte chemistry and managing dispersion for the chosen readout method are essential for optimal biosensor performance.

### 4.2. SPR-Based Fiber-Chip Platforms

SPR sensors based on optical fibers have gained increasing attention for their potential in compact, label-free, and real-time analyte detection. Recent developments have demonstrated a variety of fiber structures and fabrication techniques integrated with microfluidic systems to improve ease of use and enhance control over the sensing environment.

**Figure 7:**
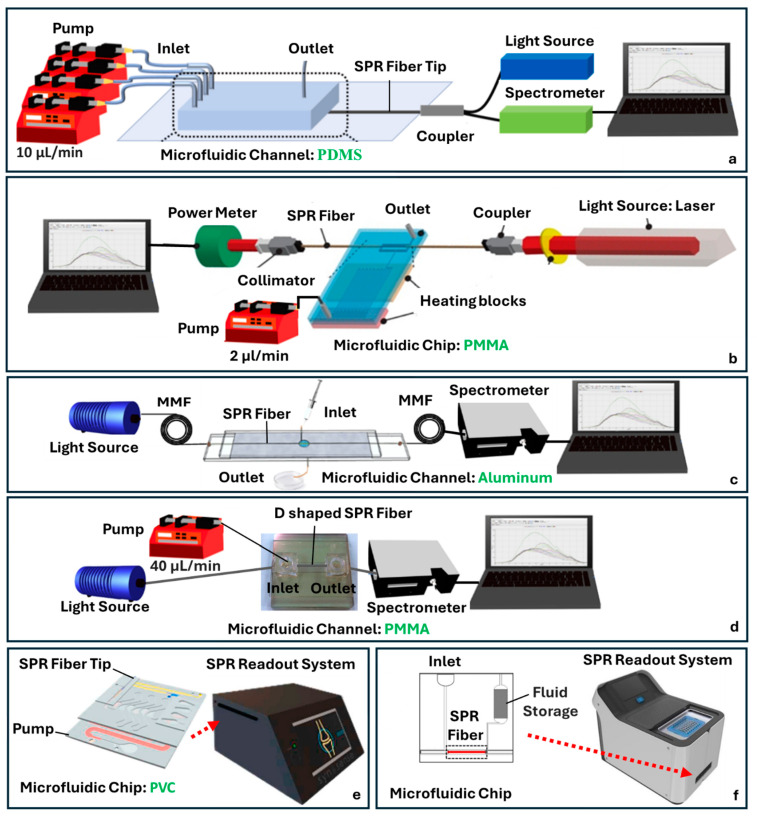
SPR-based fiber-chip platforms. (**a**) PDMS chip for thyroglobulin immunoassay on an AuNP-coated MMF tip; (**b**) PMMA PCR/SPR cartridge for real-time Salmonella DNA detection; (**c**) a Cr/Au-lined HOF embedded in an Al chip; (**d**) a curved D-shaped fiber in PMMA for ethanol breath analysis; (**e**) a self-powered PVC cartridge for adalimumab monitoring; and (**f**) a pump-free chip with an AuNP-aptamer MMF for SARS-CoV-2 nucleocapsid sensing. Optofluidic platform figures adapted from and reproduced, respectively, with permission from Refs. [55,62,63,64,65,66].(**a**–**c**,**e**) © Elsevier; (**d**,**f**) © MDPI, licensed under CC BY 4.0.

Kim et al. employed an MMF tip as a sensing interface, functionalized with AuNPs for the detection of thyroglobulin (Tg) (Figure 7a) [56]. Their approach included a microfluidic chip fabricated from PDMS, molded via soft lithography, and bonded to glass slides using oxygen plasma. The design included multiple inlets and a single outlet allowing for the sequential delivery of antibodies, analytes, and wash solutions without exposing the sensor to ambient air. This setup offered good control over reaction kinetics and minimized the risk of AuNP detachment or sample evaporation. They also incorporated a secondary amplification strategy using sandwich immunoassays, where secondary antibodies were conjugated to AuNPs and introduced after target binding to amplify the SPR signal [62]. The functionalization chemistry started with piranha-cleaned fiber tips treated with 3-Aminopropyl(diethoxy)methylsilane (APMES) to create amine groups, followed by AuNP attachment and antibody immobilization, with Bovine Serum Albumin (BSA) blocking to reduce nonspecific binding.

A different fabrication approach was taken by Nguyen et al., who integrated a polymerase chain reaction (PCR) microdevice with a bimetallic-coated SPR fiber sensor into an all-in-one PMMA-based platform (Figure 7b) [63]. The PCR channel and optical detection chamber were co-fabricated using Computer Numerical Control (CNC) milling, housing a multimode fiber coated with Ag/Aluminum (Al). This configuration enabled the real-time, label-free detection of DNA amplicons from Salmonella, with BSA passivation used to reduce nonspecific adsorption inside the microchannel. The integration of PCR and optical sensing in a single chip demonstrated a streamlined diagnostic platform.

Yang et al. developed a compact HOF spliced between two MMFs (Figure 7c) [64]. The capillary’s inner surface was uniformly coated with a Chromium (Cr)/Au bilayer using vacuum thermal evaporation, forming an SPR-active region. The assembly was embedded in a laser-engraved Al microfluidic chip sandwiched between glass sheets. Similarly to Yang et al., but utilizing a different fiber structure, Sun et al. explored a SPR sensor using a curved D-shaped POF embedded within a PMMA-based microfluidic chip (Figure 7d) [65].The D-shaped fiber was side-polished to expose the core region, Au-coated, and embedded in a curved trench to extend the interaction length. The microchannel was fabricated via CO_2_ laser ablation of the PMMA and layered with double-sided adhesive tape. Ethanol detection was achieved by monitoring RI changes in exhaled breath, with light intensity shifts corresponding to ethanol presence.

Moving toward lab-on-a-chip platforms, Qu et al. introduced a fully integrated fiber optic SPR sensor for the point-of-care monitoring of adalimumab (ADM), a monoclonal antibody used in autoimmune therapy (Figure 7e) [55]. Their device combined a gold-coated optical fiber probe with a self-powered microfluidic cartridge. The probe was pre-functionalized and inserted into the cartridge, which featured a layered structure incorporating a sample loading zone, mixing channel, and detection chamber. The covalent coupling of antibodies to AuNPs allowed for a one-step immunoassay. The design required precise buffer preparation but offered advantages in portability, low sample volume (1 µL), and autonomous liquid handling. However, the multilayer assembly added fabrication complexity and slightly underestimated ADM levels compared to ELISA and lateral flow assays. Similarly focused on point-of-care diagnostics, Chang et al. developed a fiber optic SPR biosensor coupled with a power-free microfluidic chip for detecting the SARS-CoV-2 nucleocapsid protein (Figure 7f) [66]. The sensor used MMF with an unclad region coated in AuNPs, functionalized with (1-ethyl-3-(3-dimethylaminopropyl)carbodiimide)/(N-hydroxysuccinimide) (EDC/NHS)-linked ssDNA aptamers specific to the target protein. The microfluidic chip leveraged capillary and gravitational forces to drive fluid without external pumps and incorporated a porous absorbent reservoir for passive waste collection. This self-contained system allowed for rapid detection in both simulated and clinical samples and demonstrated a robust, scalable platform suitable for pandemic-responsive point-of-care testing.

### 4.3. Raman/SERS Fiber-Chip Platforms

SERS is another sensing technique that enhances the inherently weak Raman scattering of molecules adsorbed on a nanostructured metal surface, typically silver or gold by several orders of magnitude. This enhancement arises from two primary mechanisms: electromagnetic amplification due to localized surface plasmon resonances (LSPRs) and chemical enhancement from charge–transfer interactions between the analyte and the metal surface. Integrating SERS detection with microfluidic platforms further expands its use by offering precise control over fluid handling and sample confinement.

**Figure 8:**
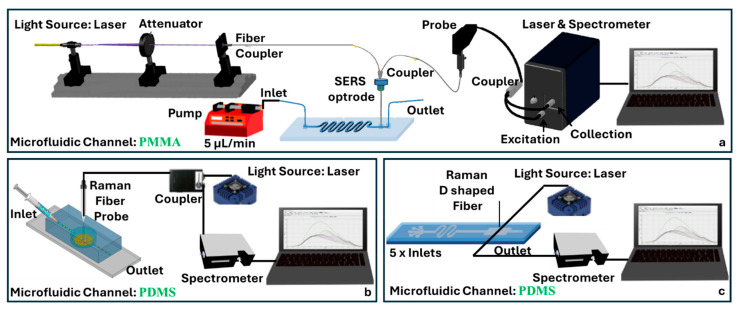
Raman/SERS fiber-chip platforms: (**a**) porous polymer-coated MMF tip within a Y-type microfluidic chip; (**b**) MOF-based SERS immunosensor in a PDMS–glass chip for BNP detection; and (**c**) AgNP-coated D-shaped MMF integrated in a PDMS chip for multiplexed sensing. Optofluidic platform figures adapted from and reproduced, respectively, with permission from Refs [59,67,68]. *©* 2014, American Chemical Society; © Elsevier; © Optica Publishing Group, respectively.

Wang et al. developed an optical fiber-based SERS by fabricating a 3D porous polymer layer directly onto the tip of an MMF (Figure 8a) [67]. Using light-initiated polymerization of glycidyl methacrylate followed by in situ AgNP growth, they created a SERS-active surface with high metal loading and effective nanoparticle aggregation for strong electromagnetic enhancement. The entire process was conducted within a microfluidic chip using a dual Y-type optical setup. The porous structure amplified the signal intensity compared to fibers modified with only AgNPs. The system achieved an LOD of 100 nM for 4-mercaptopyridine and thiram and was further demonstrated for pH sensing via protonation-responsive spectral shifts in the reporter molecule.

In contrast, Zheng et al. implemented a biologically specific SERS immunosensor for detecting brain natriuretic peptide (BNP), a key biomarker for chronic heart failure (Figure 8b) [59]. Their design utilized a sandwich immunoassay format built upon magnetic CoFe_2_O_4_- and Au- 4-(2-hydroxyethyl)-1-piperazineethanesulfonic acid (HEPES)-functionalized metal–organic frameworks loaded with toluidine blue as the Raman dye. These complexes flowed through a PDMS–glass microfluidic chip with a nanostructured SERS substrate embedded at the base. An all-fiber detection system was based on an MMF tip, a light source (laser), and a spectrometer. This system achieved a detection limit as low as 1 pg/mL for BNP. Similarly, Bo et al. developed a microfluidic-integrated D-shaped optical fiber sensor employing SERS for multiplexed molecular detection (Figure 8c) [68]. The fiber fabrication involved the mechanical polishing of MMF to achieve a D-shaped cross-section. Following polishing, the fiber surface was coated with AgNPs using a seed-mediated growth and liquid–liquid interface deposition method. The microfluidic integration was accomplished by embedding the coated fiber within a PDMS microchannel structure, formed by molding and sealed via plasma bonding. This integrated approach achieved an LOD of 0.1 nM for rhodamine 6G. The multi-channel microfluidic configuration allowed for the simultaneous detection of multiple analytes.

### 4.4. UV-Vis Absorbance Fiber-Chip Platforms

UV-Vis absorbance spectroscopy is another widely used analytical technique that quantifies analyte concentration based on the Beer–Lambert law, which relates absorbance to the product of path length, molar absorptivity, and concentration. When light passes through a sample, specific wavelengths are absorbed depending on the molecular structure of the analyte, and the resulting decrease in transmitted light intensity can be measured. Although conventional UV-Vis systems are well-established in cuvette-based formats, their application in microfluidic and lab-on-a-chip systems poses challenges due to reduced optical path lengths and detection volumes. To address this, recent research has integrated optical fibers into microfluidic platforms to achieve sensitive, low-volume absorbance detection.

**Figure 9:**
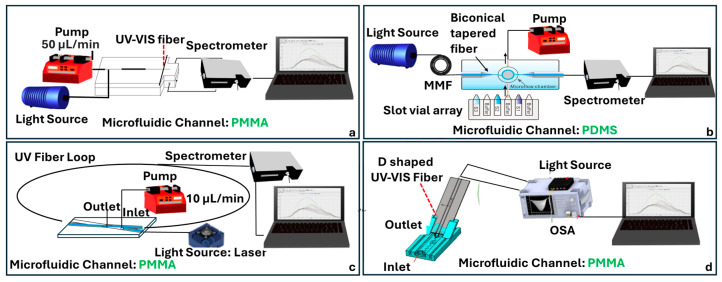
UV–Vis absorbance fiber-chip platforms: (**a**) PMMA microfluidic chip with polymerized hybrid monolith for detection of pharmaceutical compounds; (**b**) PDMS microfluidic chip with biconical coiled tapered optical fiber for detection of FeCl_3_; (**c**) UV fiber-loop embedded in PMMA plate for detection of tartrazine and myoglobin; and (**d**) U-shaped optical fiber embedded in a PMMA chip for glucose detection. Optofluidic platform figures adapted from and reproduced, respectively, with permission from Refs [18,69,70,71]. © Elsevier; © 2019, American Chemical Society; © 2009, American Chemical Society; © IEEE, respectively.

One approach to enhancing preconcentration and detection sensitivity involves embedding absorptive materials directly within the microfluidic channels. Zhang et al. developed a UV-curable organic–inorganic hybrid monolith composed of poly(MAA-co-EGDMA) and γ-Al_2_O_3_ NPs, polymerized in situ within a PMMA microfluidic chip (Figure 9a) [69]. This solid-phase microextraction column was coupled with an optical fiber UV–Vis spectrophotometer operating at 230 nm to detect 2-amino-4-chlorophenol in pharmaceutical formulations. The hybrid monolith provided an increased surface area and stability compared to polymer-only monoliths.

To overcome the limited path length inherent in microchannels, other researchers have exploited optical nanofiber geometries. Mei et al. introduced a coiled optical nanofiber wound around a PDMS pillar and integrated within a microfluidic chip (Figure 9b) [70]. Operating in the UV-Vis region, the system relied on evanescent field absorption, where a significant portion of light propagates outside the fiber and interacts with the surrounding fluid. This geometry increased the effective path length while maintaining a small footprint, achieving a 10-fold sensitivity improvement compared to standard 1 cm cuvette measurements. The device reached detection limits of 10 μM for FeCl_3_ and sub-ng/L for chloramphenicol using an enzyme-linked immunosorbent assay (ELISA), while preserving signal reversibility and throughput.

A related strategy employs cavity-enhanced techniques such as fiber-looping-down spectroscopy (FLRDS), which decouples sensitivity from absolute light intensity. Loock et al. demonstrated a 405 nm FLRDS system using UV-transparent fibers and a 190 µm sample gap with a detection volume of just 6 nL (Figure 9c) [71]. By monitoring the decay time of circulating light pulses instead of absolute absorbance, the system eliminated the need for intensity calibration and enabled the robust detection of trace analytes such as tartrazine (LOD 5 µM), myoglobin, and pharmaceutical ingredients. The ring-down time was modulated by the analyte’s absorbance in the gap region, and phase-shift techniques allowed for detection without fast electronics. This method is particularly advantageous for highly miniaturized microfluidic devices and capillary-based separations.

More recently, Hsu et al. integrated a U-shaped optical fiber into a thermally bonded PMMA microfluidic system for the detection of glucose concentrations (Figure 9d) [18]. The U-shaped fiber configuration enhanced sensitivity by promoting Whispering-Gallery Mode formation and mode interference, with a strong evanescent field generated in the bend region. The system showed consistent spectral redshifts and signal dips with increasing glucose concentration, achieving a wavelength sensitivity of 0.114 nm/% and a transmission loss sensitivity of 0.09 dB/%. Importantly, the microchannel design included zigzag geometries to improve fluid mixing and ensure accurate glucose quantification. With a strong repeatability and linear response, the sensor exemplifies a compact, low-cost approach for noninvasive glucose monitoring in biomedical diagnostics.

### 4.5. Fluorescence Fiber-Chip Platforms

Fluorescence-based detection has long served as a foundational technique in bioanalytical sensing due to its compatibility with molecular labeling, ability to monitor dynamic processes in real time, and broad applications across chemical and biological assays. When integrated with microfluidic platforms and optical fibers, fluorescence detection systems gain added benefits in terms of compactness, mechanical stability, and the ability to perform low-volume, localized analyses. However, several challenges remain, particularly background interference, signal loss, and the need for precise optical alignment. Recent advances in optofluidic integration aimed to address these limitations by embedding optical components directly into microfluidic environments.

**Figure 10:**
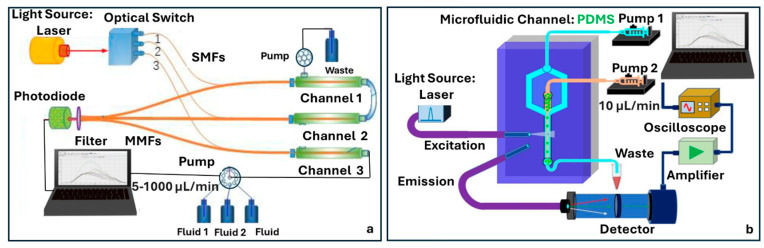
Fluorescence fiber-chip platforms: (**a**) optofluidic multiplexed assays; (**b**) single-bead analysis on PDMS microfluidic channel. Optofluidic platform figures adapted from and reproduced, respectively, with permission from Refs. [72,73]. Li et al., © Elsevier; Fattah et al., © Wiley-VCH, respectively.

Multiplexed detection has been introduced by Li et al., incorporating a fiber optical switch and a series of single-multimode couplers [72]. This modular design allowed sequential excitation and detection across multiple microchannels using a single detector. Fabricated with an emphasis on simplicity and alignment-free operation, the structure eliminated the need for lenses or mirrors, favoring fully enclosed optofluidic integration. The device also enabled automated sample handling through a pump-controlled system, making it particularly suitable for repetitive or multiplexed assays (Figure 10a). Al Fattah et al. introduced an alternative architecture based on orthogonal fiber alignment, embedding both excitation and collection multimode optical fibers at 45° angles to a microfluidic channel (Figure 10b) [73]. In this design, a fabricated PDMS microfluidic channel through soft lithography with integrated grooves for fiber placement was optimized for single-bead analysis. The use of hydrodynamic focusing ensured a consistent bead alignment within the excitation zone, while the orthogonal geometry minimized background scatter and reduced the system’s reliance on external optics. A key strength of this approach is its modularity: the plug-and-play fiber design and open access microchannel layout allow for the flexible reconfiguration and adaptation to different assay formats.

## 5. Tapered Optical Fibers

### 5.1. Fabrication Methods

**Figure 11:**
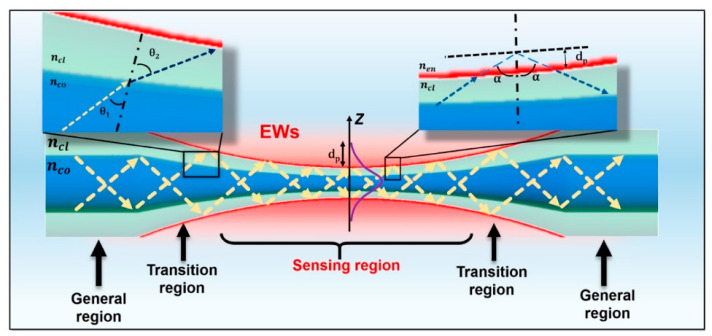
Schematic of basic tapered fiber structure adapted from Ref. [74] with permission © 2023 by the authors. Licensed under CC BY 4.0.

Tapered optical fibers are fibers whose diameters are deliberately reduced in a specific region, forming a waist that is considerably narrower than the original core and cladding. This reduced waist substantially enhances the evanescent wave, thus permitting greater interaction between the guided light and the surrounding medium (Figure 11). As a result, tapered optical fibers exhibit a heightened sensitivity to RI or absorbance changes, making them well-suited for biological and chemical sensing [26].

Tapered optical fiber fabrication techniques are broadly categorized into etching-based and heat-and-pull-based methods. Etching-based approaches, particularly wet chemical etching with HF, offer a relatively low-cost route to producing fine-tip or ultra-thin tapered structures without the mechanical stretching required by heat-and-pull methods. In several demonstrations [75,76,77,78], buffered HF has been used to selectively remove the cladding in a controlled manner, yielding tapered sections with diameters spanning from tens of micrometers down to sub-micron scales. One investigation formed a 19 µm waist subsequently coated with tin oxide for enhanced humidity sensing [77], and similar protocols have produced a 61.5 µm taper while preserving fiber strength for temperature measurements [76]. More recently, an SMF was etched to 52 µm, then gold-coated for RI detection in blood, where resonance shifts correlated with the hemoglobin concentration [75]. Although etching requires careful process control to avoid over-etching or fiber breakage, it remains a cost-effective technique for creating taper waists in optical fiber biosensors.

**Figure 12:**
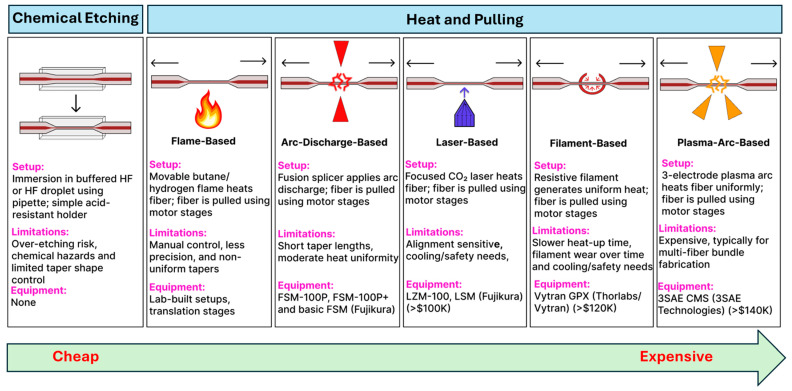
Overview of tapered optical fiber fabrication methods categorized into chemical etching and heat-and-pull techniques. Heat-and-pull methods include flame-based, arc-discharge, laser-based, and plasma-arc-based approaches, varying in precision, complexity, and cost. Setup, limitations, and representative equipment are summarized along with a cost gradient from low to high.

Heat-and-pull techniques rely on localized heating and mechanical drawing and can be further categorized by the type of heat source employed. Flame-based methods, often considered the most accessible, use an oxyhydrogen flame, sometimes in a simple butane torch setup, to soften the fiber while motorized stages pull from both ends [79,80,81]. By adjusting the flame temperature, fiber pulling speed, and flame scanning speed, researchers can precisely tune the taper geometry and waist diameter [7,82,83,84], all while minimizing surface roughness for low-loss waveguiding [74]. In arc-discharge tapering, fusion splicers (e.g., FSM100P, FSM100P+ from Fujikura) generate a controlled electric arc to heat and taper the fiber [85,86], although basic splicer models have a limited tapering functionality available [87,88]. Laser-based systems (e.g., LZM from Fujikura) offer a high-end solution, typically using a focused CO_2_ laser beam for localized heating during fiber pulling, which yields highly uniform tapers with excellent reproducibility [89,90,91,92,93,94,95]. Similarly, filament-based systems (e.g., Vytran GPX from Thorlabs) employ a thin, high-temperature filament (tungsten or platinum) to soften the fiber, allowing motorized stages to draw it into a controlled taper [4,96,97,98]. This approach is well-known for producing low-loss adiabatic tapers. At the top end of the spectrum, plasma-arc machines (e.g., 3SAE from 3SAE Technologies) rely on a high-voltage electric discharge to create a stable plasma that precisely heats a small region of the fiber [99,100,101,102,103,104], making them particularly well-suited for complex taper profiles and advanced fiber architectures such as multicore fibers [105,106,107]. More recently, an unconventional heat-and-pull method has been demonstrated in which hot water is used to soften POFs prior to tapering [108]. This approach offers a low-cost, safe, and controlled alternative to traditional heating sources, though its use has so far been limited to perfluorinated graded-index fibers (PFGIFs) and remains unexplored. Figure 12 provides an overview of both chemical etching and heat-and-pull methods, highlighting typical setups, key limitations, and representative equipment for each technique along a cost gradient from low to high. Across all methods, reproducibility depends on precisely controlling the waist radius, transition length, and taper profile to maintain an adiabatic local taper rate; deviations from adiabaticity increase scattering losses and induce modal beating. Chemical etching is low cost and capable of producing sub-micron waists but is prone to over-etching, surface roughness, and loss of tensile strength; process stability can be improved with buffered chemistries, controlled temperature/agitation, in situ optical monitoring, and post-fabrication recoating or embedding. Flame-based pulling is accessible and flexible but sensitive to gas flow, temperature drift, and flame geometry; mass-flow control, flame scanning, and clean handling might improve uniformity. Arc-discharge tapering delivers repeatable results for short hot zones but is less suited to long adiabatic profiles and may induce local residual stress; lower arc currents, multiple passes, and post-recoating might help. Laser-based pulling provides precise, stable heating and highly uniform tapers but requires costly equipment and careful alignment; a closed-loop power control and optimized scan profiles can mitigate these issues. Filament-based systems offer uniform heating for low-loss adiabatic tapers but have a slower thermal response and filament degradation; current stabilization, pre-baking, and inert atmospheres might reduce variability. Plasma-arc machines produce extremely localized, uniform heating suited for complex and multicore tapers, though at a high cost and with high-voltage safety considerations; cycle control and staged pulls might reduce the risk of microcracks. For all methods, minimizing handling, applying protective recoating or embedding, and reporting geometric parameters with uncertainties and yields are critical for ensuring mechanical strength, reproducibility, and fair comparison between methods.

### 5.2. Designs and Configurations of Tapered Optical Fiber Based Biosensors

Recent progress in tapered optical fiber biosensors shows a clear shift toward more specialized, highly sensitive designs. Researchers are relying on both basic tapered fiber waist regions [4,79,84,91,96,99] (Table 1), as well creating more sophisticated structures, such as dual-tapered segments [97], asymmetric tapers [97], tapered tips [81,98,100], and hybrid [85,88,101,102,103,109,110] and advanced fiber configurations [2,86,93,94,95,104,105,106,107,111] (Table 2). These geometries benefit from stronger evanescent fields, abundant surfaces for functionalization, and easy integration of nanoparticles [79,99,101,102,103,104,106,111], metal oxides [91,102,103,111], or two-dimensional materials [80,93,98,105,106] to boost sensitivity. At the same time, fresh approaches to surface chemistry, ranging from polyamidoamine (PAMAM) dendrimer coatings [96] to antibody [88,102,103,109] and enzyme-based recognition layers [84,91,98,99,101], have significantly improved specificity and detection limits. Designs that employ LSPR [79,99,101,102,103,104,106,111], ring laser cavities [4,110], or reflectometric methods [81,98,100] now achieve ultranarrow resonances and high signal-to-noise ratios, further enhancing sensitivity. While many systems still use straightforward dip-and-read protocols, recent examples have begun incorporating microfluidic channels to regulate sample flow [85], indicating the technology’s move toward real-world usage. The field is transitioning from proof-of-concept research to robust platforms suitable for point-of-care diagnostics and other high-impact applications [112].

Below, we explore in greater detail the specific SMF, MMF, and hybrid SMF–MMF–multicore fibers (MCF) and advanced tapered fiber geometries, their respective surface functionalization strategies, and the optical interrogation methods that have enabled a broad range of biosensing applications. Each section highlights the underlying sensing mechanisms, whether they exploit RI shifts, LSPR, or spectroscopic enhancements, and discusses how different engineering choices in taper design impact sensitivity, specificity, and practical usability. Each configuration offers unique performance characteristics. Tapered SMFs provide a stable single-mode profile, reduced modal noise, and narrow spectral features, making them particularly well-suited for high-precision, wavelength- or phase-resolved sensing modalities such as RI, LSPR tracking, and interferometry. Tapered MMFs enable a high optical throughput, larger functionalization surface area, and efficient excitation/collection in fluorescence and absorbance detection. Still, they are more prone to modal dispersion and noise, which can limit spectral resolution. Hybrid SMF–MMF–MCF configurations combine the clean spectral response of SMF with the high collection efficiency of MMF or multicore designs, offering flexibility for applications requiring both sensitivity and robust signal acquisition. Advanced tapered geometries engineer local field enhancements and customized mode profiles to maximize light–analyte interactions and broaden functionalization options, thereby further increasing sensitivity.

#### 5.2.1. Tapered SMF Geometries

A prominent application involves a 12 µm-waist SMF fabricated (Table 1a) via filament-based heat-and-pull (Vytran GPX-3000), then functionalized with PAMAM dendrimers to detect the dengue virus envelope E protein. This multi-step chemical modification, sodium hydroxide (NaOH) hydroxylation, (3-Aminopropyl)triethoxysilane (APTES) silanization, glutaraldehyde (GA) activation, and dendrimer coating yielded a LOD 1 pM in phosphate-buffered saline (PBS) by simply immersing the tapered region into the sample [96]. Optical interrogation was performed using a broadband source (1520–1620 nm) and an optical spectrum analyzer (OSA) at each fiber end to track wavelength shifts.

Another tapered SMF platform integrated the taper into a fiber ring laser (FRL) cavity to detect the avidin protein [4]. Fabricated via a filament-based heat-and-pull process (Vytran GPX-3400), the SMF featured a 15 µm waist diameter over a 10 mm taper length. After NaOH hydroxylation, silane-polyethylene glycol (PEG)-biotin was applied to capture avidin selectively. Placing the tapered SMF in line with an erbium-doped fiber (EDF), isolator, polarization controller, and wavelength division multiplexer allowed for narrow-linewidth interrogation and a high signal-to-noise ratio, although the exact LOD was not reported. This straightforward dip-and-read approach showed how ring laser amplification can increase sensor performance. A different study used LSPR to detect ascorbic acid [99]. Here, the SMF was tapered using a combination of plasma-arc (CMS 3SAE) and arc-discharge (FSM-100) systems, then coated with green-synthesized AgNPs. The multi-step surface functionalization included piranha cleaning, silanization with (3-mercaptopropyl), trimethoxysilane (MPTMS), and immobilization of ascorbate oxidase via EDC/NHS coupling. The optical setup used a tungsten-halogen light source (200–2000 nm) and a spectrometer (200–1000 nm), capturing wavelength shifts from 110 µM to 400 µM ascorbic acid. The sample solution was simply pipetted onto the tapered region placed on a glass slide. Another LSPR-based configuration employed AuNPs on a flame-pulled SMF, featuring a 10 µm waist diameter and a 1 mm taper length [79]. Piranha solution and APMES were used to prepare the surface, enabling the electrostatic immobilization of citrate-stabilized AuNPs. While the study focused primarily on simulation, it presented a practical immobilization protocol and demonstrated how binding events might shift the transmission spectrum due to local RI changes. Finally, polarization-maintaining fibers have also been adapted for heart disease biomarker detection, particularly triacylglycerides [91]. Using a laser-based (LZM100) pulling process, researchers created a tapered region, then coated it with poly(sodium-4-styrenesulfonate), followed by the growth of zeolitic imidazolate framework-8 (ZIF-8). Lipase enzyme was subsequently immobilized atop the porous ZIF-8 structure, enabling hydrolysis of triacylglycerides in a simple dip-and-read format. A broadband light source (covering 1525 nm) and an OSA monitored the resulting wavelength shifts, achieving a 0.23 nM LOD and selectivity against urea, glucose, ethanol, and ascorbic acid.

In one approach, Shaimerdenova et al. [92] incorporated magnesium oxide (MgO) NPs into an SMF taper (waist diameters: 28–33 µm; taper length: ~5 mm) to detect the cancer biomarker CD44 (Table 1b). Fabricated using a laser-based (LZM-100) heat-and-pull method, the tapered region underwent piranha cleaning, (3-Aminopropyl)trimethoxysilane (APTMS) silanization, GA activation, antibody immobilization, and mPEG-amine blocking. Detection relied on Rayleigh backscattering measured by an optical backscatter reflectometer (OBR), yielding an LOD of 16.4 pM in serum. However, specificity was limited, as controls without an antibody and with an unrelated antibody (anti-HER2) still responded at about 24% of the CD44 signal. This result highlights the importance of refining surface chemistry and expanding validation against multiple biomarkers.

In another study [25], a tapered SMF with a 50 µm waist diameter and a 0.7 m taper length was prepared via a flame-based heat-and-pull procedure, then coated with PDMS combustion products and GO to detect the cancer biomarker MUC1 (Table 1c). This sintered PDMS layer not only increased the available surface area for bioreceptor attachment but also enhanced the mechanical protection of the fragile taper. The fiber surface was further treated with NaOH, followed by APTES silanization, layer-by-layer GO deposition, EDC/NHS activation, and antibody immobilization, culminating in a 0.11 pM LOD in PBS. Low nonspecific binding was demonstrated against antibodies such as IgA, IgM, LF, and Hb, and the biosensor could be regenerated through GO antibody layer renewal. A supercontinuum light source and an OSA monitored spectral shifts, while solutions were applied via static drop-casting.

Zulkeflee et al. [97] designed a dual-tapered SMF biosensor for the simultaneous detection of the dengue II envelope (E) and SARS-CoV-2 spike (S) proteins (Table 1d). Fabricated by a flame-based heat-and-pull method, the two tapered sections (waist diameters of 12 µm and 10 µm, lengths of 0.5 cm and 2 cm, respectively) produced distinct spectral responses. After NaOH treatment, the surface was silanized with APTES and activated by GA, although neither EDC/NHS coupling nor blocking steps were included. Using a broadband C-band source (1530–1610 nm) and an OSA, the sensor achieved an impressive 0.1 pM LOD for both targets. However, specificity testing was confined to MERS-CoV as a single non-target control. All antigen-handling steps were performed manually in U-groove acrylic holders, signaling the need for broader validation and potential fluidic integration.

Chu et al. [81] developed a tip-based SMF biosensor for cancer biomarker MUC1 detection, formed by a flame-based heat-and-pull process and followed by a Au film sputtered onto the cleaved fiber end (Table 1e). Although the exact taper geometry was not reported, a reference probe with a matching free spectral range established a Vernier effect scheme that amplified the RI sensitivity by 8.4×. Piranha solution, APTES silanization, GA crosslinking, and aptamer immobilization (with BSA blocking) completed the surface functionalization. Operated in a straightforward dip-and-read format, the reflective tip sensor reached an LOD of 0.012 pg/mL for MUC1 in PBS and exhibited minimal cross-reactivity when tested against five non-target proteins. By offering high sensitivity in a compact, reflection-based design, the tip configuration lends itself well to in situ detection scenarios and small-volume clinical applications. There are additional tapered SMF-based biosensors that have been developed for various biomarkers recently, all using similar fabrications, detection mechanisms, and optical setup configurations [114,115].

#### 5.2.2. Tapered MMF Geometries

Building on the same principle of enhanced evanescent fields, tapered MMFs have also been explored for diverse biochemical sensing applications. A notable demonstration comes from Aziz et al. [84], who developed a flame-based method to fabricate a tapered MMF (Table 1f) with a 22.60 µm waist diameter. They sequentially functionalized the taper via NaOH hydroxylation, followed by APTES silanization, and finally immobilized glucose oxidase (GOx). Operating in a simple dip-and-read configuration, the sensor monitored wavelength shifts in the transmission spectrum when immersed in glucose solutions (10–50% concentration). A tungsten-halogen source (200–1000 nm) coupled to a spectrometer captured these shifts, yielding a sensitivity of 5.01 × 10^−3^ a.u./% and displaying strong selectivity over other sugars. Although the LOD was not reported, the system maintained stability for at least 20 min, highlighting the practicality of tapered MMF platforms.

Further enhancing design flexibility, Idris et al. [98] devised a tapered MMF tip sensor (Table 1g), reduced to a 50 µm waist diameter over a 10 mm tapered region, then coated with a pyrrole/polyvinyl alcohol (Py/PVA) matrix containing GOx. This coating was formed through a gamma irradiation process that simultaneously polymerized pyrrole, crosslinked PVA, and immobilized GOx. With a tunable 1550 nm light source and an optical power meter, the team monitored reflectance changes in a straightforward dip-and-read setup, confirming the sensor’s selectivity for glucose over fructose, sucrose, uric acid, and ascorbic acid.

In another example of tip-based design, Li et al. [100] produced a dual-mode sensor by partially coating a plasma-arc-pulled (CMS 3SAE) MMF tip (~30 µm waist) with a thin Ag film. This reflective plasmonic surface enabled simultaneous SERS and fluorescence detection. Using a 785 nm laser for SERS and a UV LED for fluorescence, Rhodamine 6G was detected down to 0.2 µM (SERS) or 2.25 µM (fluorescence). Like the other MMF sensors mentioned, it employed a dip-and-read approach by directly immersing the tapered tip into the analyte solution, reinforcing the value of simple sample handling in tandem with high analytical sensitivity.

#### 5.2.3. Hybrid SMF–MMF–MCF Structures

Hybrid fiber systems combine the best qualities of different fiber types, like the low-loss, single-mode performance of SMFs with the robust, multimodal characteristics of MMFs and MCFs. By carefully splicing these fibers together (FSM 100P+) and then applying precise tapering techniques (such as plasma-arc, laser-based, or filament-based heat-and-pull methods), researchers attempted to create a sensing platform that delivers much stronger evanescent fields and improved light–analyte interactions. This approach also allowed for the integration of advanced nanomaterials and specialized surface chemistries, which can improve both sensitivity and selectivity. Although the fabrication process is more complex, the enhanced performance in applications like clinical diagnostics, environmental monitoring, and food safety makes these hybrid systems a worthwhile upgrade.

For instance, one study [85] developed a SPR-based MMF-tapered SMF-MMF biosensor (Table 2a) by incorporating AuNPs to enhance the sensitivity for BSA detection. In their approach, a SMF was spliced between two MMFs and then tapered using an arc-discharge-based (FSM100P+) heat-and-pull technique (taper waist not specified). The tapered fiber was subsequently coated with an Al_2_O_3_-Ag-Au multilayer to stimulate SPR and improve the penetration depth of surface plasmon waves. To further enhance sensitivity, AuNPs (40 nm) were introduced to amplify the LSPR signal. This configuration enabled the detection of BSA with an LOD of 0.3 ng/mL. The biosensor was regenerated by washing with NaOH to break the antigen–antibody interaction, restoring the transmission spectrum while maintaining a stable performance across multiple cycles with negligible degradation. The optical path included a halogen light source and a spectrometer. The metal-coated tapered fiber was encapsulated in a PDMS microfluidic chip to improve stability, usability, and fluid control.

As a further design [109], an SMF-tapered MCF–SMF biosensor (Table 2b) was developed for C-reactive protein (CRP) detection by splicing a 15 mm quad-core MCF fiber (7.5 µm cores arranged in an equilateral triangle within a 125 µm cladding) between two SMFs and tapering it down to approximately 8.22 µm using a plasma-arc-based (3SAE) heat-and-pull technique. After KOH hydroxylation, silanization, EDC/NHS activation, and antibody immobilization with BSA blocking, the sensor achieved a 0.83 pg/mL LOD in PBS, and it showed comparable performance in blood samples, distinguishing CRP from the N-protein, TNF-α, and IgG. A broadband source (1250–1650 nm) and an OSA were used for transmission monitoring. In another configuration [88], a SMF-tapered MCF–SMF configuration targeted TNF-α by splicing a 20 mm quad-core fiber between two SMFs and tapering it to around 5.45 µm, then functionalizing the surface with a specialized silane, EDC/NHS, and TNF-α antibodies (with BSA blocking). An EDF laser integrated into the setup narrowed the output spectrum’s full width at half maximum (FWHM) from 2.85 nm to 0.08 nm, enabling a 10 pg/mL detection limit for TNF-α in blood. The interrogation system employed a 980 nm pump, erbium-doped fiber, and an OSA. Another study [101] tackled creatinine sensing with a seven-core fiber spliced between two SMFs, then tapered to a 40 µm waist via a plasma-arc-based (3SAE) technique. The tapered section was piranha-cleaned, silanized with MPTMS, and coated with 10 nm AuNPs plus Nb_2_CTₓ MXene, before EDC/NHS-mediated enzyme immobilization. Light from a tungsten-halogen lamp (HL 1000) passed through the structure, and a USB2000+ spectrometer measured the transmitted spectrum in a dip-and-read format. This LSPR sensor showed an 86.12 µM LOD for creatinine, with a high specificity against sarcosine, ascorbic acid, pyruvate acid, and uric acid.

Another innovative design [110] developed a laser ring cavity biosensor MMF-tapered MCF–MMF sensor (Table 2c) for detecting des-γ-carboxy prothrombin (DCP), a critical biomarker for hepatocellular carcinoma. In their design, gold nanorods (GNRs) with a length of 102 nm and a diameter of 10 nm were immobilized onto the fiber probe to excite LSPR at near-IR wavelengths. Integration of an EDF ring laser reduced the output spectrum’s FWHM from 2.85 nm to 0.11 nm, thereby improving the detection sensitivity and lowering the LoD to 367.6 pg/mL. In their approach, an MCF was spliced between two MMF and then tapered using a plasma-arc-based (3SAE) heat-and-pull technique. The sensor was operated in a simple dip-and-read format. A similiar approach [103] developed an LSPR MMF-tapered MCF–MMF biosensor for acetylcholine detection by incorporating AuNPs (~10 nm) and MoS_2_ nanoparticles to increase the sensitivity. In their approach, an MCF was spliced between two MMFs and then tapered using a plasma-arc-based (3SAE) heat-and-pull technique to achieve a tapered waist of 40 μm. The tapered fiber was sequentially functionalized: it was first cleaned with piranha solution to hydroxylate the surface, then silanized with MPTMS for enhanced nanoparticle adhesion. Next, a layer of AuNPs was immobilized, followed by the deposition of MoS_2_ nanoparticles, and finally, acetylcholine-specific antibodies were attached. The sensor was operated in a simple dip-and-read format, where the probe was directly immersed in the analyte solution. The optical path comprised a tungsten-halogen light source (HL-1000) and a USB2000+ spectrometer to capture transmitted spectral shifts. Additionally, control experiments with potential interfering biomolecules, such as ascorbic acid, glucose, dopamine, and uric acid, confirmed that the sensor exhibits a high specificity for acetylcholine, achieving an LOD of 14.28 μM

Finally, in one study [102], an LSPR biosensor targeting acrylamide detection in food safety was realized by splicing two MMFs to an MCF, then performing three sequential tapers (Table 2d) using a plasma-arc-based (3SAE) heat-and-pull technique to reduce waist diameters to 60, 50, and 40 µm. The tapered region was functionalized with a 10 nm AuNP, multiwalled carbon nanotubes (MWCN), and zinc oxide nanowires to increase the effective antibody-binding surface. The immobilization protocol involved piranha cleaning, MPTMS silanization, nanocomposite coating, mercaptoundecanoic acid (MUA) treatment, EDC/NHS activation, and antibody attachment, followed by a final milk powder blocking step. During measurement, a tungsten-halogen source (HL-1000) launched light through the sensor, while a USB2000+ spectrometer tracked resonance wavelength redshifts in a dip-and-read format. With the probe immersed directly in the sample, the sensor achieved a 0.438 µg/mL LOD in PBS and demonstrated robust selectivity against potential interferents such as glycine, glucose, L-cysteine, L-alanine, and sarcosine.

#### 5.2.4. Advanced Tapered Design

Advanced tapered optical fiber biosensors are evolving into highly engineered systems that merge various fiber geometries with state-of-the-art fabrication techniques to achieve superior sensitivity and specificity. These sensors are typically fabricated using highly controlled heat-and-pull methods, such as plasma-arc (e.g., 3SAE) or laser-based (e.g., LZM100) techniques, that allow for the precise control over parameters like waist diameter, taper length, and transition profiles.

One such study [2] developed a taper-in-taper (Table 2e) fiber optic biosensor for detecting CRP. In their approach, an SMF was tapered using a flame-based (AFBT-8000) heat-and-pull technique. The tapered fiber (waist 7um, length 2mm) was sequentially functionalized: it was first cleaned with piranha solution to hydroxylate the surface, then silanized with APTES, activated with GA, followed by immobilization of anti-CRP antibodies and finally blocked with BSA. The sensor was operated in a simple dip-and-read format, where the probe was fixed onto a grooved glass slide using UV-curable adhesive and directly immersed into the analyte solution. The optical path consisted of a broadband near-IR light source and an OSA to capture transmitted spectral shifts. The biosensor achieved an LOD of 0.278 μg/mL and demonstrated selectivity against BSA, human IgG, and ovalbumin. Similarly, another taper-in-taper design [111] targeted p-cresol detection for applications in aquaculture, marine life, and healthcare. In this case, a tapered SMF with a 40 µm waist over 2 mm was sequentially functionalized, beginning with piranha cleaning, followed by MPTMS silanization, deposition of AuNPs and copper oxide nanoflowers (CuO-NFs), 11-Mercaptoundecanoic acid (MUA) treatment, and immobilization of the anti-creatinase enzyme. Operated in a simple dip-and-read format using a broadband tungsten-halogen light source and spectrometer, the sensor achieved an LoD of 0.14 mM and demonstrated selectivity against uric acid, L-alanine, β-cyclodextrin, glycine, and glucose. Another advanced system [104] for liver injury monitoring employed an LSPR taper-in-taper biosensor to detect alanine aminotransferase. Here, an SMF was tapered via a plasma-arc-based (3SAE) heat-and-pull technique to produce a 40 µm waist over 2 mm. After piranha cleaning, the fiber was silanized with MPTMS and coated with AuNPs along with molybdenum disulfide (MoS_2_) and cerium oxide (CeO_2_) nanoparticles. Subsequent MUA treatment, EDC/NHS activation, and enzyme immobilization allowed the sensor, operated in a dip-and-read format with a broadband tungsten-halogen source and spectrometer, to achieve an LoD of 10.61 U/l with good selectivity against glycine, ascorbic acid, acetone, and glucose. A similar approach was also applied for detecting creatinine but instead using zinc oxide (ZnO) nanoparticles [118].

Additional innovative designs include a “humanoid” shaped SMF taper (Table 2f) [105] for histamine detection, where a plasma-arc-based (3SAE) technique produced dual waist diameters (40 and 80 µm over a 1 mm length), followed by sequential functionalization (piranha cleaning, GO and MWCNT deposition, MPTMS, AuNPs, MUA, EDC/NHS activation, and enzyme immobilization). This configuration, operated in a dip-and-read format, yielded an LoD of 59.45 μM and selectivity against tryptamine, spermine, tyramine, putrescine, and L-α-alanine.

Similarly, an S-shaped tapered MMF with an SMF ball (Table 2g) [106] was fabricated via plasma-arc-based tapering (3SAE) to produce a sensor with waists of 40 and 80 µm over 1 mm. Following piranha cleaning, MPTMS treatment, deposition of AuNPs, MWCNTs combined with Nb_2_CTₓ, MUA modification, EDC/NHS activation, enzyme immobilization, and a BSA blocking step, this sensor achieved an LOD of 0.267 μM for putrescine, demonstrating selectivity against HSA, BSA, SPE, and tryptophan. A similar fabrication methodology and detection mechanism were recently applied for detecting putrescine at LOD 0.8223 μM [119].

Moreover, periodically tapered SMFs (Table 2h), comprising between 5 and 45 tapered sections with waist diameters from 12 to 110 µm, have been developed for detecting analytes such as pepsin [93], hemoglobin [94,95], prostate-specific antigen (PSA) [86], and ascorbic acid [107]. These systems typically involve an initial NaOH hydroxylation, followed by APTES silanization and subsequent coating with graphene oxide (GO) or a polydopamine–GO composite, combined with EDC/NHS coupling for bioreceptor immobilization. Optical interrogation in these devices is carried out with a broadband light source and either an OSA or spectrometer to capture transmitted spectral shifts.

**Figure 13:**
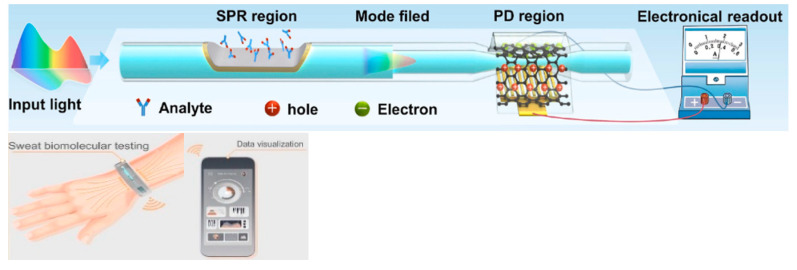
Schematic representation of the integrated sensor structure. Integrated devices are employed [112].

Tapered optical fibers can function not only as biosensing elements but also as efficient transducers for converting optical signals into electrical outputs. In a recent study by Shen et al., a tapered SMF (waist diameter: 40 μm, length 2 mm) was fabricated using a flame-based technique [112]. A graphene protected by a polymethyl methacrylate (PMMA) layer on a tapered fiber was used as a photodetector. This region was strategically positioned to receive light signals from a side-polished fiber SPR sensor, which detected changes in RI due to glucose and urea in artificial perspiration. The evanescent field leaking from the tapered SMF excited photogenerated carriers in the graphene layer. These carriers were then collected by interdigitated Au electrodes fabricated on a glass substrate beneath the fiber. The resulting photocurrent was measured using a digital source meter, allowing the device to convert optical intensity changes into quantifiable electrical signals. This design demonstrated that the tapered SMF not only transmitted the SPR signal but also enabled direct electronic readout, effectively replacing bulky OSA or spectrometers and enabling a compact, integrated, and wearable biosensing system (Figure 13).

### 5.3. Outside-Fiber Optofluidics Utilizing Tapered Fibers

From an engineering standpoint, the fiber acts merely as an insert, so the chip substrate can be chosen to fit the application: glass for low autofluorescence, PDMS for rapid prototyping and self-sealing around the fiber, or thermoplastics for high-throughput injection molding. This decoupling facilitates easy replacement or multiplexing; multiple coated fibers can be arrayed in parallel channels for multi-analyte panels or swapped out when fouled. Finally, outside-fiber formats are geometry-agnostic: flat-end, D-shaped, side-polished, or long-taper sections can all be embedded, allowing SPR, SERS, and absorbance or fluorescence to be realized within a single fabrication paradigm.

**Figure 14:**
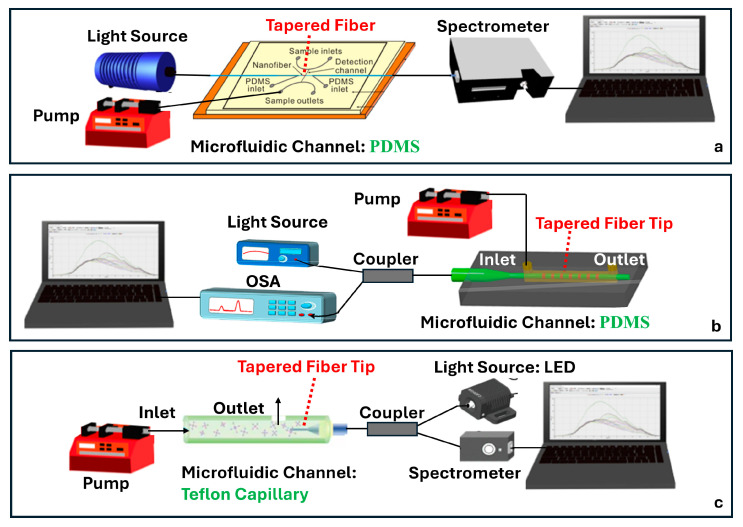
Outside-fiber optofluidics utilizing tapered fibers. (**a**) Tapered fiber; (**b**) tapered grating assisted fiber tip; and (**c**) tapered fiber ball tip. Optofluidic platforms adapted and reproduced with permission from Refs. [113,116,117]. © OSA, and Sun et al. (2014) © Elsevier. (2022) © IEEE, respectively.

Several studies published before 2025 have laid the groundwork for integrating tapered optical fibers with outside-fiber optofluidics, offering practical and effective strategies to enhance sensitivity, reduce sample volume, and achieve compact sensor designs. When embedded in microfluidic environments, the tapered fibers benefit from directed sample flow, stable interaction zones, and reduced optical losses due to improved analyte confinement. For example, Zhang et al. [113] describe a configuration using a 0.8 μm-waist tapered SMF placed perpendicularly across a 5 μm-wide PDMS microchannel (Figure 14a). The exposed tapered region created a ~1.0 femtoliter detection volume, enabling both fluorescence and RI sensing with real-time spectral acquisition and fast response (~600 ms).

The microfluidic chip offered several advantages, including a compact footprint, low sample consumption, and also made this platform especially well-suited for the analysis of high-concentration biological samples with extremely limited sample volumes. In another study by Sun et al., a tapered MMF tip was functionalized for DNA hybridization sensing and integrated into a PDMS chip [117]. The Bragg grating inscribed on the tapered MMF tip enabled in situ detection with a detection limit of 0.5 μM and low reagent use (~50 μL) (Figure 14b). The PDMS microfluidic system offered a compact and efficient sensing platform for a fragile tapered tip (taper waist: 4 µm), significantly reducing reagent consumption while maintaining high detection sensitivity. Li et al. [116] focused on the fluorescence detection of aggregation-induced emission molecules using a tapered MMF ball tip fiber embedded in a Teflon capillary. The microfluidic system was built using a Teflon AF2400 capillary that acted as a liquid-core waveguide, confining and directing a small sample volume (0.649 μL) over the sensitive region (Figure 14c). This integration directed the sample fluid precisely over the sensitive fiber tip, thereby optimizing analyte delivery and enhancing sensor performance. In a different study, a 7.2 μm-waist tapered SMF was embedded in a PDMS microchannel fabricated using SU-8 lithography [120]. The setup demonstrated low detection limits (100 pM to 10 pM) for fluorescent dyes and quantum-dot-labeled proteins, using a controlled sample flow to minimize background and improve reproducibility. Song et al. reported a dual-color sensing system that used a 200 μm-waist tapered fiber and a fiber optic switch to alternate between two excitation wavelengths [121]. This platform achieved 2 amol and 20 amol detection limits for Cy5.5 and pacific blue, respectively, using a silicon-based photodetector, which improved integration and reduced the cost. Another approach developed a PDMS-based microfluidic channel for encapsulating an MMF-tapered SMF-MMF construct for the detection of BSA (LOD of 0.3 ng/mL) [85]. Their method involved using a 125 µm diameter optical fiber as a mold for forming the PDMS channel. Specifically, the optical fiber was fixed onto a silicon wafer to create the cylindrical microchannel mold. After pouring and curing the PDMS, the fiber mold was carefully removed, leaving behind a precisely aligned microchannel within the PDMS substrate. Two holes were then punched at opposite ends of the channel to serve as inlet and outlet ports. The PDMS was bonded to a glass slide using oxygen plasma treatment to form a sealed microfluidic channel. Finally, the MMF-tapered SMF–MMF was carefully inserted into the microchannel, and epoxy adhesive was used to seal the channel ends, thereby securing the tapered fiber in place within the microfluidic platform for high-sensitivity analyte detection. This approach was straightforward, avoiding the need to fabricate custom molds, and resulted in a channel geometry that closely matched the optical fiber dimensions. As a result, the fluidic volume surrounding the fiber was minimized (not specified), with the final device having a fiber diameter of approximately 150 µm. Table 3 summarizes key design parameters and performance outcomes for each of these studies.

## 6. Challenges and Benefits of Fiber-Based Optofluidic Biosensors

In-fiber optofluidics integrate the optical waveguide and microfluidic channel within the same fiber, enabling continuous light–analyte interactions over long propagation distances, which yields a high sensitivity, efficient use of ultra-low sample volumes (µL–nL), and reagent economy [122]. The tight confinement of light in the fiber core enhances signal accumulation and supports real-time monitoring. However, fabrication is technically demanding: tapering hollow fibers, femtosecond laser micromachining, or splicing microstructured fibers must preserve both optical guiding and fluidic sealing [123]. Hollow-core fibers are inherently fragile, and current designs often depend on external pumps, light sources, and detectors, limiting portability. Outside-fiber optofluidics embed the sensing fiber into an external microfluidic chip, where analytes interact with the fiber’s outer surface. This simplifies fabrication, provides mechanical stability from chip housing, and accommodates specialized fiber geometries, such as tapered, D-shaped [12,68], U-shaped [18], and side-polished, that enhance evanescent-field coupling and sensitivity. Tapered fibers, in particular, extend the evanescent field’s reach and, when combined with microfluidic confinement, improve binding rates, detection speed, and measurement stability [116,124,125,126,127,128], while also allowing for the integration of mixers, filters, and reference channels [22,129]. Surface functionalization in outside-fiber formats is straightforward, supporting selective detection via nanoparticles, thin films, or biorecognition elements [68,71], and multiple fibers can be integrated into one chip for multiplexed sensing [71,73]. Plasmonic or dielectric coatings on tapered regions can further enhance sensitivity, though they may degrade over time, a limitation that controlled microfluidic environments can help mitigate [19,130].

In addition to these performance-related considerations, real-world deployment requires attention to recyclability, cost, and durability. Recyclability depends on the fiber substrate and surface chemistry. Silica and certain polymer fibers can be regenerated through cleaning and refunctionalization, though repeated use may gradually reduce optical performance or biorecognition efficiency [131]. Cost drivers include the choice between standard telecom-grade fibers and specialty designs (e.g., photonic crystal, hollow-core), fabrication complexity (tapering, etching, and nanostructuring), and the expense of functionalization reagents. For instance, optical fiber biosensors are generally seen as fragile and high cost in interrogation methods, although recent approaches are increasingly robust and cost-effective [132]. Economies of scale can significantly reduce per-unit costs, such as for fiber processing, standardized connectors, or integration with molded microfluidic platforms. Durability hinges on resistance to fouling, photobleaching, environmental exposure, and mechanical stress; strategies to enhance longevity include protective cladding, robust chip housings, and chemically stable surface chemistries.

## 7. Pathways to Self-Contained Fiber-Based Optofluidic Biosensors

Advancing in-fiber and outside-fiber optofluidic biosensors toward fully self-contained “lab-on-a-fiber” platforms requires integrated solutions that minimize reliance on external fluid handling and optical instrumentation. For in-fiber systems, strategies such as capillary-driven flow using hydrophilic inner surfaces [123], integration of on-fiber light sources (micro-LEDs, chip-scale lasers) and detectors, and the incorporation of reagent reservoirs or porous scaffolds for on-fiber sample preparation, can reduce auxiliary equipment needs and enhance portability. Outside-fiber configurations can exploit on-chip microvalves, electroosmotic pumps, and paper-based microfluidics for compact, low-power fluid control, along with additive manufacturing to produce transparent microfluidic chips embedding fiber grooves and reservoirs in a single step. Tapered fibers within microfluidics can benefit from protective packaging that preserves their enhanced sensitivity while enabling plug-and-play operation. Multifunctional designs that combine plasmonic, dielectric, or 2D nanomaterial coatings with microfluidic confinement can increase performance, while regenerative or anti-fouling surfaces support reusability [19,130]. For both configurations, integrating compact power sources, wireless data transmission, and embedded electronics for signal processing and machine learning-based interpretation will enable real-time, remote, and multiplexed biosensing in point-of-care, environmental, or wearable applications. Collectively, these developments can help to transition high-performance laboratory prototypes to robust, autonomous fiber optic diagnostic tools.

## 8. Non-Fiber Microfluidic Sensing Platforms and Comparison with Fiber-Based Systems

Non-fiber-based microfluidic platforms generally employ chip- or substrate-integrated detection elements fabricated from glass, silicon, or polymers (e.g., PDMS), where sensing is achieved through optical, electrical, or mechanical transduction. Examples include dielectrophoretic sensors, which use non-uniform AC electric fields to manipulate, concentrate, or separate target particles or cells to improve sensitivity [133]; spectroscopic “spectral tweezers,” which combine optical trapping with Raman, fluorescence, or absorption/reflection spectroscopy for label-free biochemical profiling at the single-particle or single-cell level [134,135]; and electrochemical impedance spectroscopy devices, which employ microfabricated electrodes within the channel to monitor impedance changes associated with biomolecular binding or cell–surface interactions [136]. Other notable chip-based approaches include surface acoustic wave devices for label-free mechanical sensing [137] and microcantilever arrays for mass or viscosity detection [138]. In contrast, fiber-based microfluidic biosensors exploit the optical fiber either as an integral part of the microchannel (e.g., optofluidic integration where the tapered or microstructured fiber forms the sensing region inside the channel) or as an external probe inserted into the microfluidic path, using the fiber’s evanescent field or interferometric modes to interrogate the sample. This allows fiber-based systems to combine the micro-scale flow control of microfluidics with the inherent advantages of optical fibers, remote and distributed sensing capabilities, immunity to electromagnetic interferences, small footprints, and compatibility with a range of detection modalities such as RI sensing, LSPR, fluorescence, and interferometry [19,139]. Compared to non-fiber-based platforms, fiber-integrated systems generally excel in field-deployable, in situ, and harsh-environment applications. In contrast, chip-based microfluidics offer higher throughput, and more extensive on-chip integration of actuators and electronics. While both classes of platforms address similar biosensing goals, the choice between them is ultimately dictated by application priorities.

## 9. Conclusions

Tapered optical fiber optofluidic sensors represent a mutually enhancing marriage between tapered fibers with microfluidic platforms, offering combined advantages such as low detection limits, high specificity, compatibility with small sample volumes, autonomous or passive flow control, and improved robustness. This review has provided a comprehensive overview of optofluidic sensors, examining both inside- and outside-fiber configurations, with a particular emphasis on the potential of tapered optical fibers in these systems. Inside-fiber optofluidic platforms leverage the internal structure of the fiber as both a light guide and fluidic channel, allowing extended analyte–light interaction lengths that enhance sensitivity. However, these systems are often constrained by fabrication complexity, reduced mechanical stability, and limited compatibility with modular or integrated architectures, such as lab-on-a-fiber systems. In contrast, outside-fiber platforms offer a more practical and robust approach, with easier fiber handling, improved mechanical stability, and more effortless adaptability with surface modification and functionalization techniques. These advantages have led to a broader focus in recent research on outside-fiber architectures, which support a variety of detection mechanisms, including SPR, SERS, absorbance, and fluorescence within microfluidic environments.

Tapered optical fibers, when compared to standard fibers, offer significantly enhanced evanescent fields and stronger light–analyte interactions, making them highly effective for biosensing applications. Their geometry allows for precise surface functionalization, further increasing detection sensitivity. This review has explored various tapered fiber designs, ranging from basic SMF tapers to advanced configurations such as SMF/MMF composites and taper-in-taper structures, each contributing unique advantages to sensing performance. Despite their potential, tapered fibers are inherently fragile and highly sensitive to mechanical and environmental conditions. As such, integrating them into outside-fiber microfluidic platforms provides not only mechanical protection but also environmental stability, supporting more reliable and repeatable sensing. Although the combination of tapered fibers with outside-fiber microfluidic systems remains relatively underexplored, early studies show their promise for high-sensitivity and low-volume biosensing. Future research should focus on advancing these platforms through the incorporation of multiplexed sensing capabilities, passive flow control (e.g., capillary action), and full integration of optical components such as micro-LEDs or on-chip photodetectors. Additionally, embedding microcontrollers for signal processing and wireless communication would enable the transition from bench-top demonstrations to fully autonomous, portable lab-on-a-fiber systems. With continued research in microfabrication techniques, tapered optical fiber optofluidic devices stand as a promising avenue for next-generation point-of-care diagnostics and wearable monitoring technologies.

## Figures and Tables

**Table 1:** Overview of reported tapered SMF and MMF biosensor geometries and configurations. The table summarizes cladding/core diameters, taper waist dimensions, optical path setups, and sample handling/delivery methods: (a) standard tapered SMF; (b) tapered asymmetric doped SMF; (c) tapered SMF coated with polydimethylsiloxane (PDMS); (d) dual-tapered SMF; (e) tapered SMF tip; (f) standard tapered MMF; (g) tapered MMF tip; (h) tapered MMF ball tip; and (i) tapered MMF tip grating inscribed.

	Taper Type	Cladding/Core Diameter (μm)	Taper Waist Diameter (μm) Taper Waist Length (mm)	Optical Path Setup	Fluidic Flow Setup	Refs.
a	Tapered SMF	125 μm/ 8–9 μm	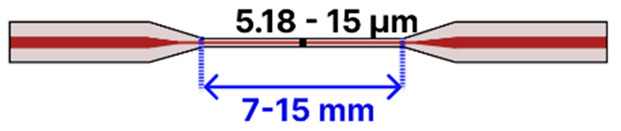	Light Source → Tapered Fiber → OSA/Spectrometer	Drop-casting, immersion/ Microfluidic Platform	[4,79,96,99,113,114,115]
b	Tapered Asymmetric Doped SMF	125 μm/ 15 μm	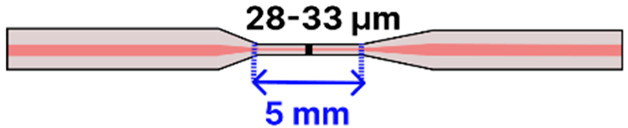	Backscatter Reflectometer ↔ Tapered Fiber	Drop-casting, immersion	[92]
c	Tapered PDMS Coated SMF	125 μm/ 9 μm	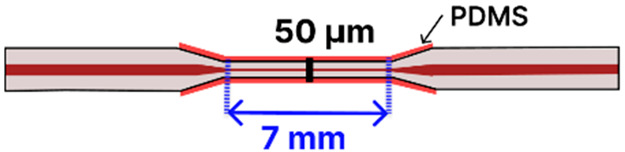	Light Source → Tapered Fiber → OSA	Drop-casting, immersion	[80]
d	Dual-Tapered SMF	125 μm/ 8.2 μm	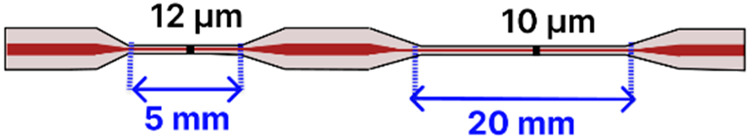	Light Source → Tapered Fiber → OSA	Drop-casting, immersion	[97]
e	Tapered SMF Tip	125 μm/ NA	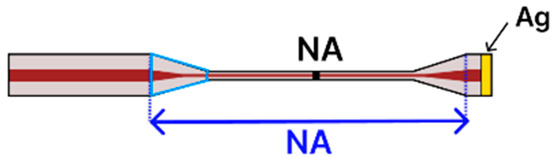	Light Source → Tapered Fiber → OSA	Dipping, immersion	[81]
f	Tapered MMF	125 μm/ 60 μm	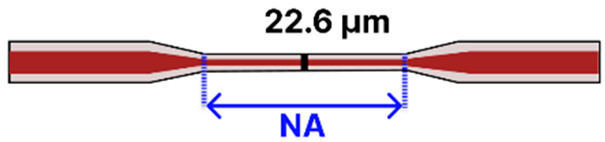	Light Source → Tapered Fiber → Spectrometer	Drop-casting, immersion	[84]
g	Tapered MMF Tip	125 μm/ 62.5–105 μm	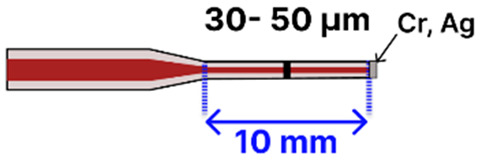	Light Source → Tapered Fiber → Power Meter/Raman Spectroscopy/Spectrometer	Dipping, immersion	[98,100]
h	Tapered MMF Ball Tip	125 μm/ 105 μm	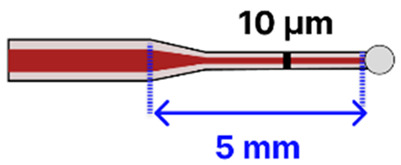	Light Source → Tapered Fiber → Spectrometer	Microfluidic Platform	[116]
i	Tapered MMF Tip Grating Inscribed	125 μm/ 62.5 μm	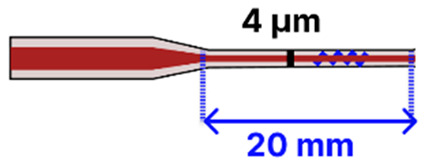	Light Source/Spectrometer ↔ Tapered Fiber	Microfluidic Platform	[117]

**Table 2:** Overview of reported hybrid and advanced SMF, MCF, and MMF biosensor geometries and configurations: (a) MMF-tapered SMF–MMF, (b) SMF-tapered MCF–SMF, (c) MMF-tapered MCF–MMF, (d) triple-tapered MCF–MMF, (e) taper-in-taper (SMF), (f) SMF-tapered humanoid, (g) SMF ball with S-shaped tapered MMF, and (h) periodically tapered SMF. The table summarizes cladding/core diameters, taper waist dimensions, optical path setups, and sample handling/delivery methods.

	Taper Type	Cladding/Core Diameter (μm)	Taper Waist Diameter (μm) Taper Waist Length (mm)	Optical Path Setup	Fluidic Flow Setup	Refs.
a	MMF-Tapered SMF-MMF	125 μm/ SMF: 8 μm MMF: 62.5 μm	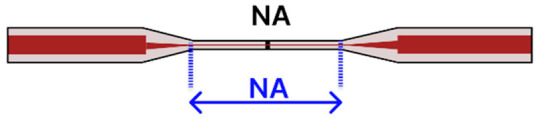	Light Source → Tapered Fiber → Spectrometer	Microfluidic Platform	[85]
b	SMF—Tapered MCF—SMF	125 μm/ SMF: 8–9 μm MCF: 6.1–7.5 μm	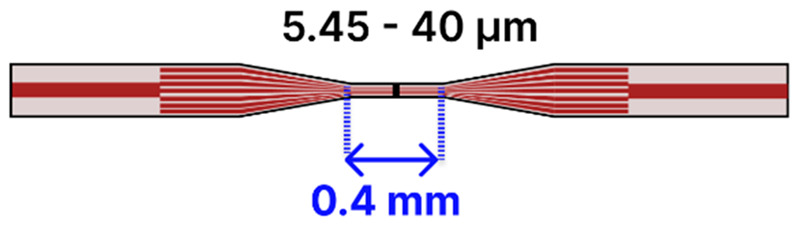	Light Source → Tapered Fiber → OSA/Spectrometer	Drop-casting, immersion	[88,101,109]
c	MMF—Tapered MCF—MMF	125 μm/ MMF: 62.5 μm MCF: 6.1 μm	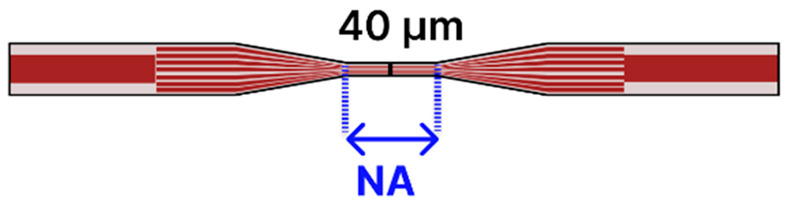	Light Source → Tapered Fiber → Spectrometer	Drop-casting, immersion	[103,110]
d	MMF—3 Tapered MCF—MMF	125 μm/ MMF: 62.5 μm MCF: 6.1 μm	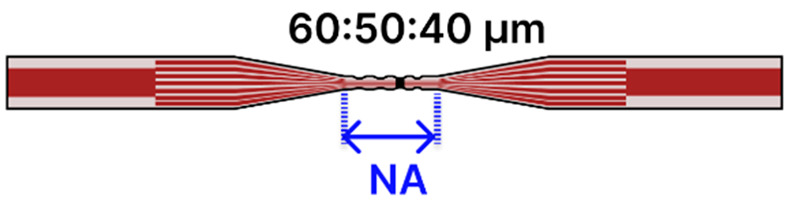	Light Source → Tapered Fiber → OSA/Spectrometer	Drop-casting, immersion	[102]
e	Taper-in-Taper	125 μm/ 9 μm	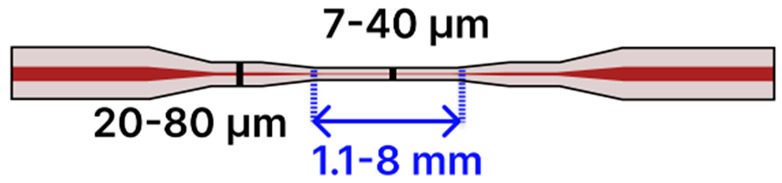	Light Source → Tapered Fiber → OSA/Spectrometer	Drop-casting, immersion	[2,104,111,118]
f	SMF Tapered Humanoid	125 μm/ 8.2 μm	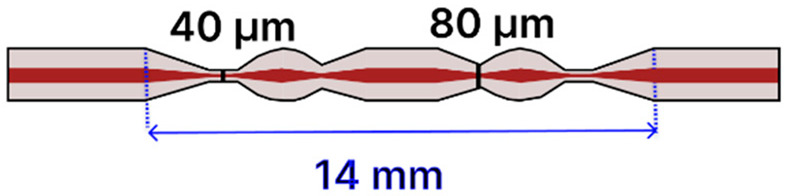	Light Source → Tapered Fiber → Spectrometer	Drop-casting, immersion	[105]
g	SMF Ball: S-Shaped Tapered MMF	125 μm/ SMF: 8.2 μm; MMF: 62.5 μm	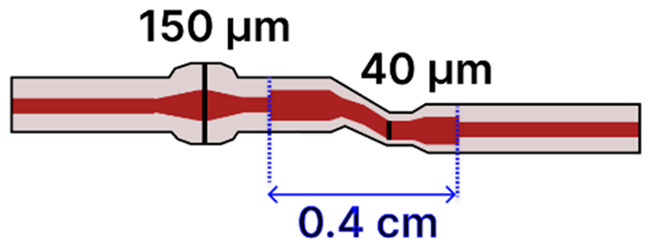	Light Source → Tapered Fiber → Spectrometer	Drop-casting, immersion	[106,119]
h	Periodically Tapered SMF	125 μm/ ~8–10 μm	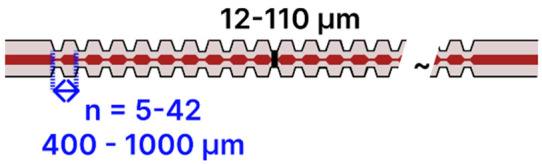	Light Source → Tapered Fiber → OSA	Drop-casting, immersion	[86,93,95,107]

**Table 3:** Summary of outside-fiber optofluidic-tapered fiber sensor studies.

Fiber Type and Taper	Microfluidic Platform	Sample Volume	Analytes	LOD	Optical Setup	Key Features	Ref.
Tapered SMF, ~0.8 µm waist (flame-based tapering)	PDMS chip, 5 µm wide detection channel	~1.0 fL	Fluorescein RI sensing	Fluorescence: 10^−7^ M RI sensing: 2.8 × 10^−4^ RIU	Fluorescence: 473 nm laser → Tapered SMF → CCD microscope RI sensing: Broadband unpolarized tungsten-halogen lamp → Tapered SMF → Spectrometer	Femtoliter detection, fast response (~600 ms)	[113]
Tapered MMF Tip (125 µm/62.5 µm), ~4 µm waist (flame-based tapering + Bragg grating)	PDMS chip, Width 200 µm × Height 150 µm microchannel	~50 µL	Target DNA (hybridization)	0.5 µM	Broadband light source/Spectrometer ↔ Tapered MMF Tip	Bragg grating, label-free DNA sensing, and partial reusability	[117]
Tapered MMF Ball Tip (125 µm/105 µm), ~10 µm waist (plasma-arc-based tapering)	Teflon AF2400 capillary (229 µm ID)	0.649 µL	TPE-COOH	0.316 µM	UV LED (340 nm)/CCD-type spectrometer ↔ Tapered MMF ball tip	Ball tip fiber, laser-ablated flow port, and optimized analyte delivery	[116]
Tapered SMF, ~7.2 µm waist (flame-based tapering)	PDMS chip, Width 125 µm × Height 150 µm × Length 5 cm	~500 nL	Rhodamine 6G, QD-Streptavidin	100 pM (R6G), 10 pM (QD-Strep)	532 nm laser/Spectrometer ↔ Tapered SMF	Soft lithography, controlled flow, and minimized nonspecific binding	[120]
Tapered MMF, 200 µm waist (HF etching)	Custom microfluidic cell (35 mm × 800 µm × 800 µm)	N/A	Cy5.5, Pacific Blue	2 aM (Cy5.5), 20 aM (PB)	Laser diode (405 nm/635nm) → Tapered MMF → Silicon-based photodetector	Dual-color, fiber optical switch, and low-cost SOP-1000 photodetector	[121]

## Data Availability

Data are contained within the article.

## References

[1] Taha B.A., Ali N., Sapiee N.M., Fadhel M.M., Mat Yeh R.M., Bachok N.N., Al Mashhadany Y., Arsad N. (2021). Comprehensive Review Tapered Optical Fiber Configurations for Sensing Application: Trend and Challenges. Biosensors.

[2] Xiao L., Chen X., Li X., Zhang J., Wang Y., Li D., Hong X., Shao Y., Chen Y. (2025). Enhanced Sensitivity Mach–Zehnder Interferometer-Based Tapered-in-Tapered Fiber-Optic Biosensor for the Immunoassay of C-Reactive Protein. Biosensors.

[3] Raghuwanshi S.K., Ansari M.T.I., Shadab A. (2024). Analysis of Tapered Fiber-Optic Surface Plasmon Resonance (SPR) Bio-Sensing Probe With the Effect of Different Taper Profiles and Metal Choices. IEEE Trans. Plasma Sci..

[4] Mansor M., Abu Bakar M.H., Omar M.F., Mustapha Kamil Y., Zainol Abidin N.H., Mustafa F.H., Mahdi M.A. (2020). Taper biosensor in fiber ring laser cavity for protein detection. Opt. Laser Technol..

[5] Mumtaz F., Roman M., Zhang B., Huang J. (2023). Assembly-free ultra-sensitive miniaturized strain sensor based on an asymmetric optical fiber taper. Measurement.

[6] Liyanage T., Lai M., Slaughter G. (2021). Label-free tapered optical fiber plasmonic biosensor. Anal. Chim. Acta.

[7] Yang X., Zhang H., Zhang H., Hou L., Yang J. (2024). Sensitivity-Enhanced Fiber-Optic Axial-Strain Sensor by Tapered-Microfiber-Assisted Micro-Open Cavity. IEEE Trans. Instrum. Meas..

[8] Khalaf A.L., Mohamad F.S., Abdul Rahman N., Lim H.N., Paiman S., Yusof N.A., Mahdi M.A., Yaacob M.H. (2017). Room temperature ammonia sensor using side-polished optical fiber coated with graphene/polyaniline nanocomposite. Opt. Mater. Express.

[9] Zhao J., Cao S., Liao C., Wang Y., Wang G., Xu X., Fu C., Xu G., Lian J., Wang Y. (2016). Surface plasmon resonance refractive sensor based on silver-coated side-polished fiber. Sens. Actuators. B Chem..

[10] Li H., Chu R., Cao J., Zhou F., Guo K., Zhang Q., Wang H., Liu Y. (2023). Sensitive and reproducible on-chip SERS detection by side-polished fiber probes integrated with microfluidic chips. Measurement.

[11] Yang M., Liu H., Zhang D., Tong X. (2010). Hydrogen sensing performance comparison of Pd layer and Pd/WO3 composite thin film coated on side-polished single- and multimode fibers. Sens. Actuators B Chem..

[12] Zhang Y., Yi Z., Shi Y., Liu C., Li X., Lv J., Yang L., Chu P.K. (2022). Photonic fibre crystal sensor with a D-shape based on surface plasma resonance containing microfluidic channels for detection of a wide range of refractive indexes. J. Mod. Opt..

[13] Lidiya A.E., Raja R.V.J., Pham V.D., Ngo Q.M., Vigneswaran D. (2019). Detecting hemoglobin content blood glucose using surface plasmon resonance in D-shaped photonic crystal fiber. Opt. Fiber Technol..

[14] Ahmed K., Paul B.K., Vasudevan B., Rashed A.N., Maheswar R., Amiri I.S., Yupapin P. (2019). Design of D-shaped elliptical core photonic crystal fiber for blood plasma cell sensing application. Results Phys..

[15] Wu X., Wang Y., Zhang J., Zhang Y., Rao X., Chen C., Liu H., Deng Y., Liao C., Smietana M.J. (2023). A D-Shaped Polymer Optical Fiber Surface Plasmon Resonance Biosensor for Breast Cancer Detection Applications. Biosensors.

[16] Kadhim R.A., Salih Q.M., Hasan A.D., Alkhasraji J.M.D., Kalankesh H.V. (2024). D-Shaped Microfluidic Channel Bimetallic with a Highly Sensitive SPR RI Sensor for a Large Detection Range. Plasmonics.

[17] Chen L.-Q., Wu Y.-C., Liu Y., Cai H.Y., Liu J. (2024). Highly sensitive dual-function sensor for refractive index and temperature using D-shaped microchannel photonic crystal fiber. Opt. Express..

[18] Hsu H.-C., Tran T.-B., Nguyen T.T.-V., Le H.-D., Chiang C.-C. (2024). Integration of a Microfluidics System With a U-Shaped Optical Fiber Sensor for Sensing Various Concentrations of Glucose. IEEE Sens. J..

[19] Wang W., Xia L., Xiao X., Li G. (2024). Recent Progress on Microfluidics Integrated with Fiber-Optic Sensors for On-Site Detection. Sensors.

[20] Xia L., Li G. (2021). Recent progress of microfluidics in surface-enhanced Raman spectroscopic analysis. J. Sep. Sci..

[21] Li L., Zhang Y., Zhou Y., Zheng W., Sun Y., Ma G., Zhao Y. (2021). Optical Fiber Optofluidic Bio-Chemical Sensors: A Review. Laser Photon. Rev..

[22] Blue R., Uttamchandani D. (2016). Recent advances in optical fiber devices for microfluidics integration. J. Biophotonics.

[23] Kumar A., Subrahmanyam T.V.B., Sharma A.D., Thyagarajan K., Pal B.P., Goyal I.C. (1984). Novel refractometer using a tapered optical fibre. Electron. Lett..

[24] Sidhik S., Ittiarah J.V., Gangopadhyay T.K., Lakshminarayanan V., Bhattacharya I. (2015). Design and Analysis of Chemically Etched and Biconically Tapered Fiber for Chemical Sensing Application. Advances in Optical Science and Engineering. Springer Proceedings in Physics.

[25] Tian Y., Wang W., Wu N., Zou X., Wang X. (2011). Tapered optical fiber sensor for label-free detection of biomolecules. Sensors.

[26] Korposh S., James S.W., Lee S.-W., Tatam R.P. (2019). Tapered Optical Fibre Sensors: Current Trends and Future Perspectives. Sensors.

[27] Esteban Ó., González-Cano A., Díaz-Herrera N., Navarrete M.-C. (2006). Absorption as a selective mechanism in surface plasmon resonance fiber optic sensors. Opt. Lett..

[28] Wei X., Peng Y., Chen X., Zhang S., Zhao Y. (2023). Optimization of tapered optical fiber sensor based on SPR for high sensitivity salinity measurement. Opt. Fiber. Technol..

[29] Teng C., Maosen L., Min R., Shijie D., Ming C., Minmin X., Libo Y., Hongchang D. (2022). A High-Sensitivity SPR Sensor Based on MMF-Tapered HCF-MMF Fiber Structure for Refractive Index Sensing. IEEE Sens. J..

[30] Liu Z., Ji X., Qin Y.K., Zhang Y., Mou J., Deng Y., Liu W., Zhang Y., Yuan L. (2023). Refractive index SPR sensor with high sensitivity and wide detection range using tapered silica fiber and photopolymer coating. Opt. Express.

[31] Tang J.L., Cheng S.F., Hsu W.T., Chiang T.Y., Chau L.K. (2006). Fiber-optic biochemical sensing with a colloidal gold-modified long period fiber grating. Sens. Actuators B Chem..

[32] Shang L., Cheng Y., Zhao Y. (2017). Emerging Droplet Microfluidics. Chem. Rev..

[33] Jung W., Han J., Choi J.W., Ahn C.H. (2015). Point-of-care testing (POCT) diagnostic systems using microfluidic lab-on-a-chip technologies. Microelectron. Eng..

[34] Chen H., Chen C., Bai S., Gao Y., Metcalfe G., Cheng W., Zhu Y. (2018). Multiplexed detection of cancer biomarkers using a microfluidic platform integrating single bead trapping and acoustic mixing techniques. Nanoscale.

[35] Essaouiba A., Okitsu T., Kinoshita R., Jellali R., Shinohara M., Danoy M., Legallais C., Sakai Y., Leclerc E. (2020). Development of a pancreas-liver organ-on-chip coculture model for organ-to-organ interaction studies. Biochem. Eng. J..

[36] Zhang X., Yuan T. (2025). Remarks of Optical Fibers and Devices for Microfluidic Sensing: Preparation and Processing. Photonic Sens..

[37] Wu J., Li Y., Song B., Zhang C., Wang Q., Gao X., Huang K. (2022). Microstructured Optical Fiber Based on Surface Plasmon Resonance for Dual-Optofluidic-Channel Sensing. Plasmonics.

[38] Li B.-L., Li D.-R., Chen J.-H., Liu Z.-Y., Wang G.-H., Zhang X.-P., Xu F., Lu Y. (2018). Hollow core micro-fiber for optical wave guiding and microfluidic manipulation. Sens. Actuators B Chem..

[39] Wan H., Chen J., Wan C., Zhou Q., Wang J., Zhang Z. (2019). Optofluidic microcapillary biosensor for label-free, low glucose concentration detection. Biomed. Opt. Express.

[40] Zhang H., Han B., Li X., Zhao Y., Zhang Y.N. (2024). An Optical Fiber Optofluidic Laser Biosensor for Rapid Hemoglobin Detection Using Organic Dye. J. Light. Technol..

[41] Yang X., Yuan T., Yue G., Li E., Yuan L. (2015). Optofluidic integrated in-fiber fluorescence online optical fiber sensor. Sens. Actuators B Chem..

[42] Yang X., Yu W., Liu Z., Yang J., Zhang Y., Kong D., Long Q., Yuan T., Cao J., Yuan L. (2018). Optofluidic twin-core hollow fiber interferometer for label-free sensing of the streptavidin-biotin binding. Sens. Actuators B Chem..

[43] Wang X., Li S., Gao S., Wang Y., Wang P., Ebendorff-Heidepriem H., Ruan Y. (2021). Microfluidic raman sensing using a single ring negative curvature hollow core fiber. Biosensors.

[44] Adamu A.I., Wang Y., Correa R.A., Bang O., Markos C. (2021). Low-loss micro-machining of anti-resonant hollow-core fiber with focused ion beam for optofluidic application. Opt. Mater. Express.

[45] Wang Y., Yang X., Zhang Y., Zhu Z., Wang R., Li X., Guan A., Tian F., Teng P., Gao S. (2024). Optofluidic In-Fiber Surface-Enhanced Raman Detection Based on Cloverleaf Hollow Optical Fiber. IEEE Sens. J..

[46] Merdalimova A.A., Rudakovskaya P.G., Ermatov T.I., Smirnov A.S., Kosolobov S.S., Skibina J.S., Demina P.A., Khlebtsov B.N., Yashchenok A.M., Gorin D.A. (2022). SERS Platform Based on Hollow-Core Microstructured Optical Fiber: Technology of UV-Mediated Gold Nanoparticle Growth. Biosensors.

[47] Khozeymeh F., Melli F., Capodaglio S., Corradini R., Benabid F., Vincetti L., Cucinotta A. (2022). Hollow-Core Fiber-Based Biosensor: A Platform for Lab-in-Fiber Optical Biosensors for DNA Detection. Sensors.

[48] Moeglen-Paget B., Perumal J., Humbert G., Olivo M., Dinish U.S. (2024). Optofluidic photonic crystal fiber platform for sensitive and reliable fluorescence based biosensing. Biomed. Opt. Express.

[49] Gao R., Lu D.F., Cheng J., Jiang Y., Jiang L., Xu J.D., Qi Z.M. (2016). Fiber optofluidic biosensor for the label-free detection of DNA hybridization and methylation based on an in-line tunable mode coupler. Biosens. Bioelectron..

[50] Testa G., Persichetti G., Bernini R. (2024). Optofluidic biosensing: Devices, strategies, and applications. TrAC Trends Anal. Chem..

[51] Aralekallu S., Boddula R., Singh V. (2023). Development of glass-based microfluidic devices: A review on its fabrication and biologic applications. Mater. Des..

[52] Pattanayak P., Singh S.K., Gulati M., Vishwas S., Kapoor B., Chellappan D.K., Anand K., Gupta G., Jha N.K., Gupta P.K. (2021). Microfluidic chips: Recent advances, critical strategies in design, applications and future perspectives. Microfluid. Nanofluid..

[53] Lin L., Chung C.-K. (2021). PDMS Microfabrication and Design for Microfluidics and Sustainable Energy Application: Review. Micromachines.

[54] Niu P., Jiang J., Liu K., Wang S., Jing J., Xu T., Wang T., Liu Y., Liu T. (2022). Fiber-integrated WGM optofluidic chip enhanced by microwave photonic analyzer for cardiac biomarker detection with ultra-high resolution. Biosens. Bioelectron..

[55] Qu J.-H., Ordutowski H., Van Tricht C., Verbruggen R., Barcenas Gallardo A., Bulcaen M., Ciwinska M., Gutierrez Cisneros C., Devriese C., Guluzade S. (2022). Point-of-care therapeutic drug monitoring of adalimumab by integrating a FO-SPR biosensor in a self-powered microfluidic cartridge. Biosens. Bioelectron..

[56] Kim H.-M., Jeong D.H., Lee H.-Y., Park J.-H., Lee S.-K. (2021). Design and validation of fiber optic localized surface plasmon resonance sensor for thyroglobulin immunoassay with high sensitivity and rapid detection. Sci. Rep..

[57] Yin M., Huang B., Gao S., Zhang A.P., Ye X. (2016). Optical fiber LPG biosensor integrated microfluidic chip for ultrasensitive glucose detection. Biomed. Opt. Express.

[58] Hu X.-G., Zhao Y., Peng Y., Chen X.-M., Wang L.-F., Lin Z.-T., Hu S. (2023). In-situ label-free temperature-compensated DNA hybridization detection with a fiber-optic interferometer and a fiber Bragg grating for microfluidic chip. biosens. Bioelectron..

[59] Zheng D., Wang Z., Wu J., Li S., Li W., Zhang H., Li X. (2022). A Raman immunosensor based on SERS and microfluidic chip for all-fiber detection of brain natriuretic peptide. Infrared Phys. Technol..

[60] Nielsen J.B., Hanson R.L., Almughamsi H.M., Pang C., Fish T.R., Woolley A.T. (2020). Microfluidics: Innovations in Materials and Their Fabrication and Functionalization. Anal. Chem..

[61] Scott S.M., Ali Z. (2021). Fabrication methods for microfluidic devices: An overview. Micromachines.

[62] Kim H.-M., Kim H.-J., Park J.-H., Lee S.-K. (2022). High-performance biosensor using a sandwich assay via antibody-conjugated gold nanoparticles and fiber-optic localized surface plasmon resonance. Anal. Chim. Acta.

[63] Nguyen T.T., Trinh K.T.L., Yoon W.J., Lee N.Y., Ju H. (2017). Integration of a microfluidic polymerase chain reaction device and surface plasmon resonance fiber sensor into an inline all-in-one platform for pathogenic bacteria detection. Sens. Actuators B Chem..

[64] Yang Z., Xia L., Li S., Qi R., Li X.C.W. (2019). Highly sensitive refractive index detection based on compact HSC-SPR structure in a microfluidic Chip. Sens. Actuators A Phys..

[65] Sun Y.-S., Li C.-J., Hsu J.-C. (2016). Integration of Curved D-Type Optical Fiber Sensor with Microfluidic Chip. Sensors.

[66] Chang T.-C., Sun A.Y., Huang Y.-C., Wang C.-H., Wang S.-C., Chau L.-K. (2022). Integration of Power-Free and Self-Contained Microfluidic Chip with Fiber Optic Particle Plasmon Resonance Aptasensor for Rapid Detection of SARS-CoV-2 Nucleocapsid Protein. Biosensors.

[67] Wang S., Liu C., Wang H., Chen G., Cong M., Song W., Jia Q., Xu S., Xu W.A. (2014). A Surface-Enhanced Raman Scattering Optrode Prepared by in Situ Photoinduced Reactions and Its Application for Highly Sensitive On-Chip Detection. ACS Appl. Mater. Interfaces.

[68] Bo H., Ke Y., Yong Z., Jie Z. (2023). Microfluidic integrated D-shaped optical fiber SERS probe with high sensitivity and ability of multi-molecule detection. Opt. Express.

[69] Zhang J., Chen G., Tian M., Li R., Quan X., Jia Q. (2013). A novel organic–inorganic hybrid monolithic column prepared in-situ in a microchip and its application for the determination of 2-amino-4-chlorophenol in chlorzoxazone tablets. Talanta.

[70] Mei H., Pan J., Zhang Z., Zhang L., Tong L. (2019). Coiled Optical Nanofiber for Optofluidic Absorbance Detection. ACS Sens..

[71] Waechter H., Bescherer K., Dürr C.J., Oleschuk R.D., Loock H.-P. (2009). 405 nm Absorption Detection in Nanoliter Volumes. Anal. Chem..

[72] Li W., Wang H., Yang R., Song D., Long F., Zhu A. (2018). Integrated multichannel all-fiber optofluidic biosensing platform for sensitive and simultaneous detection of trace analytes. Anal. Chim. Acta.

[73] Fattah M.A.A., Abouali H., Hosseini S.A., Yin J., Khan A.A., Aghamohammadi H., Poudineh M., Ban D. (2024). An Optofluidic System for Monitoring Fluorescently Activated Protein Biomarkers. Anal. Sens..

[74] Zhang W., Lang X., Liu X., Li G., Singh R., Zhang B., Kumar S. (2023). Advances in Tapered Optical Fiber Sensor Structures: From Conventional to Novel and Emerging. Biosensors.

[75] Raham H.S., Nida M.H. (2024). Tapered single-mode no-core optical fiber sensor coated with layer gold for hemoglobin sensing. J. Opt..

[76] Zhu X., Huang Q., Chen H., Ling Q., Ren Z., Peng B., Chen D. (2024). Ultra-high sensitive refractive index sensor based on etched SNS fiber structure and self-imaging. Opt. Commun..

[77] Son G., Jung Y., Yu K. (2017). Tapered Optical Fiber Couplers Fabricated by Droplet-Based Chemical Etching. IEEE Photonics J..

[78] Ascorbe J., Corres J.M., Matias I.R., Arregui F.J. (2016). High sensitivity humidity sensor based on cladding-etched optical fiber and lossy mode resonances. Sens. Actuators B Chem..

[79] Borjikhani P., Granpayeh N., Zibaii M.I. (2025). High sensitivity tapered fiber refractive index biosensor using hollow gold nanoparticles. Sci. Rep..

[80] Wang B., Liu S., Gao M., Li Y., Yang H., Sun C., Guo Q., Yu Y. (2025). A highly sensitive tapered fiber biosensor modified by PDMS combustion product and graphene oxide for MUC1 detection. Sens. Actuators Rep..

[81] Chu C., Yang X., Jiang H., Yang X., Ma M., Teng P., Wang S., Wang R., Wen X., Li K. (2025). Parallel tapered optical fiber biosensor for highly sensitive detection of Mucin 1. IEEE Sens. J..

[82] Kahlet N.A., Isa N.M., Alauddin S.M., Burham N., Saad H. (2024). Enhancement of Sensing Performance for Alcohol in Aqueous Solution using Tapered Optical Fiber Coated with Polyaniline via Air-Brushing Technique. Int. J. Integr. Eng..

[83] Wang H., Zhen R., Wen Z., Bao X., Cai Y., Gao S. (2024). Analysis and fabrication of dual-core tapered fiber for high-sensitivity and high-accuracy multiparameter sensing. Opt. Laser Technol..

[84] Aziz M.S., Shamsudin M.S., Fahri M.A.S.A., Syuhada A., Ibrahim R.K.R., Bakhtiar H., Harun S.W. (2022). Glucose oxidase-based enzyme immobilised on tapered optical fibre for reliability improvement in selective glucose sensing. Optik.

[85] Yang X., Guo J., Yang F., Yang G., Wu Y., Li Z., Liu Y., Yang X., Yao J. (2025). Tapered optical fiber LRSPR biosensor based on gold nanoparticle amplification for label-free BSA detection. Sens. Actuators B Chem..

[86] Xiao P., Sun Z., Huang Y., Lin W., Ge Y., Xiao R., Li K., Li Z., Lu H., Yang M. (2020). Development of an optical microfiber immunosensor for prostate specific antigen analysis using a high-order-diffraction long period grating. Opt. Express.

[87] Zhang G., Singh R., Zhang B., Kumar S., Li G. (2023). WaveFlex biosensor based on S-tapered and waist-expanded technique for detection of glycosylated hemoglobin. Biomed. Opt. Express.

[88] Cao H., Liu J., Wei D., Liu B., Hu Y., Liu J., Wan S., Wu T., He X.-D., Jiang J. (2025). Ultrahigh sensitivity fiber laser integrated biosensor for TNF-α detection in human blood sample. Sens. Actuators B Chem..

[89] Yang Q., Hu D., Li Z., Xu Z., Ran Y., Guan B. (2024). Assembly tapered fiber Bragg grating tip with gold nanostars for heat generation and gradient temperature sensing. Opt. Laser Technol..

[90] Hidayat N., Safwan Abd Aziz M., Nur H., Taufiq A., Mufti N., Rakhmata Mukti R., Bakhtiar H. (2023). Sensitivity enhancement of gold nanospheres assisted CO2 laser tapered optical fiber for refractive index sensor. Opt. Fiber Technol..

[91] Lu L., Zhu L., Zhu G., Dong M., Zeng Z. (2020). ZIF-8/Lipase Coated Tapered Optical Fiber Biosensor for the Detection of Triacylglycerides. IEEE Sens. J..

[92] Shaimerdenova M., Ayupova T., Ashikbayeva Z., Bekmurzayeva A., Blanc W., Tosi D. (2023). Reflector-Less Shallow-Tapered Optical Fiber Biosensors for Rapid Detection of Cancer Biomarkers. J. Light. Technol..

[93] Kang X., Wang R., Jiang M., Li E., Li Y., Wang T., Ren Z. (2023). A label-free biosensor for pepsin detection based on graphene oxide functionalized micro-tapered long period fiber grating. Sens. Actuators Rep..

[94] Wang R., Ren Z., Kong D., Hu B., He Z. (2020). Highly sensitive label-free biosensor based on graphene-oxide functionalized micro-tapered long period fiber grating. Opt. Mater..

[95] Li Y., Du M., He S., Wang R., Zhang Z., Wang Q. (2023). Sensitive label-free hemoglobin detection based on polydopamine functionalized graphene oxide coated micro-tapered long-period fiber grating. Optik.

[96] Kamil Y.M., Al-Rekabi S.H., Yaacob M.H., Syahir A., Chee H.Y., Mahdi M.A., Abu Bakar M.H. (2019). Detection of dengue using PAMAM dendrimer integrated tapered optical fiber sensor. Sci. Rep..

[97] Zulkeflee N.N., Kamil Y.M., Mashohor S., Bakar M.H.A. (2025). Functionalized cascaded tapered optical fiber sensor for simultaneous detection of dengue II E and SARS-CoV-2 S proteins. Biosens. Bioelectron..

[98] Idris S., Azeman N.H., Azmy N.A.N., Ratnam C.T., Mahdi M.A., Bakar A.A.A. (2018). Gamma irradiated Py/PVA for GOx immobilization on tapered optical fiber for glucose biosensing. Sens. Actuators B Chem..

[99] Gangal A., Choudhary K., Duseja M., Shukla R.K., Kumar S. (2025). Green synthesis of Silver nanoparticles from plant oil for enzyme-functionalized optical fiber biosensor: Improved sensitivity and selectivity in ascorbic acid detection. Opt. Laser Technol..

[100] Li M., Yan M., Xu B., Zhao C., Wang D., Wang Y., Chen H. (2023). A dual-mode optical fiber sensor for SERS and fluorescence detection in liquid. Spectrochim. Acta. A Mol. Biomol. Spectrosc..

[101] Li M., Singh R., Soares M.S., Marques C., Zhang B., Kumar S. (2022). Convex fiber-tapered seven core fiber-convex fiber (CTC) structure-based biosensor for creatinine detection in aquaculture. Opt. Express.

[102] Zhou X., Xie Y., Singh R., Zhang B., Kumar S. (2025). Smart Fiber-Optic WaveFlex Biosensor With Nano-Interface for Acrylamide Monitoring in Food Safety Applications. IEEE Sens. J..

[103] Zhu G., Wang Y., Wang Z., Singh R., Marques C., Wu Q., Kaushik B.K., Jha R., Zhang B., Kumar S. (2022). Localized Plasmon-Based Multicore Fiber Biosensor for Acetylcholine Detection. IEEE Trans. Instrum. Meas..

[104] Wang Z., Singh R., Marques C., Jha R., Zhang B., Kumar S. (2021). Taper-in-taper fiber structure-based LSPR sensor for alanine aminotransferase detection. Opt. Express.

[105] Zhang W., Singh R., Wang Z., Li G., Xie Y., Jha R., Marques C., Zhang B., Kumar S. (2023). Humanoid shaped optical fiber plasmon biosensor functionalized with graphene oxide/multi-walled carbon nanotubes for histamine detection. Opt. Express.

[106] Zhang Z., Li G., Li X., Singh R., Min R., Zhang B., Kumar S. (2025). Ultra-sensitive fiber optic LSPR biosensor with enhanced enzyme functionalization for real-time putrescine detection. Opt. Express.

[107] Zhu G., Agrawal N., Singh R., Kumar S., Zhang B., Saha C., Kumar S. (2020). A novel periodically tapered structure-based gold nanoparticles and graphene oxide—Immobilized optical fiber sensor to detect ascorbic acid. Opt. Laser Technol..

[108] Mizuno Y., Ujihara H., Lee H., Hayashi N., Nakamura K. (2017). Polymer optical fiber tapering using hot water. Appl. Phys. Express.

[109] Gao Q., Wei D., Yao Y., Liu B., Liu J., Hu Y., Yang H., Fu Y., Zhang Y., Wan S. (2025). Ultrahigh-Sensitivity Fiber Biosensor for C-Reactive Protein Detection in Blood Sample. IEEE Sens. J..

[110] Li X., Singh R., Zhang B., Kumar S., Li G. (2025). Ultralow-limit of detection optical fiber LSPR biosensor based on a ring laser for des-*γ*-carboxy prothrombin detection. Photonics Res..

[111] Zhang Y., Singh R., Marques C., Malathi S., Li Q., Vyoma W., Kumar S., Zhang B. (2022). Plasmon-Based Tapered-in-Tapered Fiber Structure for p-Cresol Detection: From Human Healthcare to Aquaculture Application. IEEE Sens. J..

[112] Shen C., Huang J., Hu S., Chen Y., Zhang L., Yi C., Hu X., Chen Y., Chen L., Liu G. (2025). Direct electronical readout of surface plasmon resonance biosensor enabled by on-fiber Graphene/PMMA photodetector. Biosens. Bioelectron..

[113] Zhang L., Li Z., Mu J., Fang W., Tong L. (2015). Femtoliter-scale optical nanofiber sensors. Opt. Express.

[114] González-León K., Delgado-Macuil R.J., Vertti-Cervantes B., Muñoz-Aguirre S., Castillo-Mixcóatl J., García-Juárez M., Montes-Narvaez O., Ramírez-Sánchez E., Beltrán-Pérez G. (2025). Application of support vector machine technique to optical fiber biosensors for neuroprotector (IL-10) detection in serum samples of murine model. Opt. Laser Technol..

[115] Zhang Y., Jing W., Zhang Y., Qiu S., Wang W., Guo Y., Han Y., Zu L., Jiang L., Yuan J. (2025). Label-free microfiber biosensor for ultrahigh sensitivity detection of TNF-α protein. Opt. Express.

[116] Li Y., Li M., Chen H., Wang D.N., Wang Y., Zhao C. (2022). A Microfluid Fiber Device for Trace Detection of Aggregation Induced Emission Molecules. IEEE Sens. J..

[117] Sun D., Guo T., Ran Y., Huang Y., Guan B.-O. (2014). In-situ DNA hybridization detection with a reflective microfiber grating biosensor. Biosens. Bioelectron..

[118] Liu F., Zhang W., Lang X., Liu X., Singh R., Li G., Xie Y., Zhang B., Kumar S. (2023). Development of Taper-in-Taper-Based Optical Fiber Sensors for Chemical and Biological Sensing. Photonics.

[119] Ma W., Li G., Li X., Singh R., Zhang B., Kumar S. (2025). Signal-Enhanced Fiber-Optic LSPR Sensor With Hybrid Nanointerface for Ultrasensitive Detection of Putrescine in Low Concentrations. IEEE Sens. J..

[120] Li Z., Xu Y., Fang W., Tong L., Zhang L. (2015). Ultra-sensitive nanofiber fluorescence detection in a microfluidic chip. Sensors.

[121] Song D., Yang R., Fang S., Liu Y., Liu J., Xu W., Long F., Zhu A. (2019). A novel dual-color total internal reflection fluorescence detecting platform using compact optical structure and silicon-based photodetector. Talanta.

[122] Ricciardi A., Crescitelli A., Vaiano P., Quero G., Consales M., Pisco M., Esposito E., Cusano A. (2015). Lab-on-fiber technology: A new vision for chemical and biological sensing. Analyst.

[123] Hassan S.U., Tariq A., Noreen A., Donia H., Zaidi S.Z.J., Bokhari H., Zhang X. (2020). Capillary-Driven Flow Microfluidics Combined with Smartphone Detection: An Emerging Tool for Point-of-Care Diagnostics. Diagnostics.

[124] Battat S., Weitz D.A., Whitesides G.M. (2022). An outlook on microfluidics: The promise and the challenge. Lab. Chip..

[125] Choi K., Mudrik J.M., Wheeler A.R. (2015). A guiding light: Spectroscopy on digital microfluidic devices using in-plane optical fibre waveguides. Anal. Bioanal. Chem..

[126] Hu Y., Muhammad T., Wu B., Wei A., Yang X., Chen L. (2020). A simple on-line detection system based on fiber-optic sensing for the realtime monitoring of fixed bed adsorption processes of molecularly imprinted polymers. J. Chromatogr. A.

[127] Yuan Y., Jia H., Xu D., Wang J. (2023). Novel method in emerging environmental contaminants detection: Fiber optic sensors based on microfluidic chips. Sci. Total Environ..

[128] Zhu J.M., Shi Y., Zhu X.Q., Yang Y., Jiang F.H., Sun C.J., Zhao W.H., Han X.T. (2017). Optofluidic marine phosphate detection with enhanced absorption using a Fabry–Pérot resonator. Lab. Chip..

[129] Dong R., Li Y., Liu S., Li W., Tao L., Chen C., Qian Z., Yang Y. (2022). On-chip spectroscopic monitoring of erythrocyte oxygenation under hematocrit and oxygen gradients. J. Sci. Adv. Mater. Devices.

[130] Zhao Y., Hu X., Hu S., Peng Y. (2020). Applications of fiber-optic biochemical sensor in microfluidic chips: A review. Biosens. Bioelectron..

[131] Hunt H.K., Armani A.M. (2011). Recycling microcavity optical biosensors. Opt. Lett..

[132] Leitão C., Pereira S.O., Marques C., Cennamo N., Zeni L., Shaimerdenova M., Ayupova T., Tosi D. (2022). Cost-Effective Fiber Optic Solutions for Biosensing. Biosensors.

[133] Henriksson A., Neubauer P., Birkholz M. (2022). Dielectrophoresis: An Approach to Increase Sensitivity, Reduce Response Time and to Suppress Nonspecific Binding in Biosensors?. Biosensors.

[134] Casabella S., Scully P., Goddard N., Gardner P. (2016). Automated analysis of single cells using Laser Tweezers Raman Spectroscopy. Analyst.

[135] Zaman M.A., Wu M., Ren W., Jensen M.A., Davis R.W., Hesselink L. (2024). Spectral tweezers: Single sample spectroscopy using optoelectronic tweezers. Appl. Phys. Lett..

[136] Chen Y.S., Huang C.H., Pai P.C., Seo J., Lei K.F. (2023). A Review on Microfluidics-Based Impedance Biosensors. Biosensors.

[137] Länge K., Rapp B.E., Rapp M. (2008). Surface acoustic wave biosensors: A review. Anal. Bioanal. Chem..

[138] Wang J., Xu B., Zhu Y., Zhao J. (2023). Microcantilever sensors for biochemical detection. J. Semicond..

[139] Gao S., Yang X., Wang S., Chu C., Teng P., Tian F., Zhang Y., Liu Z., Yang X. (2024). Review of Optical Fiber Optofluidic Chemical Sensors and Biosensors. Photonic Sens..

